# Transcriptomic biomarker pathways associated with death in HIV-infected patients with cryptococcal meningitis

**DOI:** 10.1186/s12920-021-00914-1

**Published:** 2021-04-16

**Authors:** Irina Vlasova-St Louis, Abdu K. Musubire, David B. Meya, Henry W. Nabeta, Hesham Mohei, David R. Boulware, Paul R. Bohjanen

**Affiliations:** 1 Department of Medicine, University of Minnesota, Minneapolis, MN USA; 2 Infectious Disease Institute, Makerere University, Kampala, Uganda

**Keywords:** Fatal immune reconstitution inflammatory syndrome, Transcriptomic biomarkers, AIDS/HIV, Cryptococcal meningitis IRIS

## Abstract

**Background:**

Cryptococcal meningitis (CM) is a major cause of death in HIV-infected patients in sub-Saharan Africa. Many CM patients experience cryptococcosis-associated immune reconstitution inflammatory syndrome (C-IRIS), which is often fatal. We sought to identify transcriptomic biomarker pathways in peripheral blood that are associated with or predict the development of death or fatal C-IRIS among patients with CM who were enrolled in the Cryptococcal Optimal ART Timing Trial.

**Methods:**

We assessed peripheral blood gene expression using next-generation RNA sequencing in 4 groups of patients with CM: (1) no C-IRIS or Death; (2) C-IRIS survivors; (3) fatal C-IRIS; (4) Death without C-IRIS. Gene expression was assessed at the time of ART initiation, at 1, 4, and 8 weeks on ART, and at the time of C-IRIS events.

**Results:**

We identified 12 inflammatory and stress response pathways, including interferon type 1 signaling, that were upregulated at the time of ART initiation in patients with future fatal C-IRIS, as compared with survivors. The upregulation of transcripts involved in innate immunity (inflammasome, Toll-like receptor signaling), was observed at the time of fatal or nonfatal C-IRIS events. At the time of fatal C-IRIS events, numerous transcripts within fMLP, Rho family GTPases, HMGB1, and other acute phase response signaling pathways were upregulated, which reflects the severity of inflammation and systemic oxidative stress. Patients who died without recognized C-IRIS also had increased expression of pathways associated with oxidative stress and tissue damage.

**Conclusions:**

Our results showed that overactivated innate immunity, involving Toll-like receptor/inflammasome pathways, and inflammation-induced oxidative stress, are associated with fatal outcomes. The results of this study provide insight into the molecular drivers of death and fatal C-IRIS to inform future diagnostic test development or guide targeted treatments.

**Supplementary Information:**

The online version contains supplementary material available at 10.1186/s12920-021-00914-1.

## Background

Cryptococcal meningitis (CM) is the most prevalent fungal opportunistic infection in persons living with AIDS with CD4 + T cell counts of less than 100 cells/uL. HIV-associated cryptococcal infections account for nearly one-fifth of the million deaths annually worldwide, with the majority occurred in the Sub-Saharan region [1]. The pathogenesis of death in CM is largely unknown. Many CM patients die due to a disseminated fungal infection that includes the involvement of the brain and improved antifungal treatment regimens are needed [2, 3]. Yet even with effective antifungal therapy, up to 20% of patients with CM die after initiating antiretroviral therapy (ART) due to cryptococcal immune reconstitution inflammatory syndrome (C-IRIS), an exaggerated and dysregulated inflammatory response that occurs as the immune system begins recovery [4]. C-IRIS often presents as clinical worsening in the setting of effective antifungal and antiviral therapy [5]. In this study, we evaluated the transcriptome in peripheral blood of patients treated for CM who survived or died after ART initiation, in order to identify biomarker pathways associated with death and better understand the molecular pathogenesis of fatal C-IRIS.

The samples used for this study were collected from patients with CM during the Cryptococcal Optimal ART Timing (COAT) trial [6]. This was a randomized clinical trial to assess survival in cryptococcal patients who received earlier ART, initiated within 1–2 weeks of antifungal therapy (early arm), or after approximately 4–6 weeks of antifungal therapy (deferred arm). The trial concluded that deferring ART for 4–6 weeks led to a 15% improvement in survival [6]. The incidence of C-IRIS did not differ significantly between the earlier-ART arm and the deferred-ART arm. However, mortality from C-IRIS appeared to be higher when ART was initiated earlier [6, 7].

In this study, we performed longitudinal transcriptomic analyses using next-generation RNA sequencing on whole blood samples from patients with CM, who survived or died from C-IRIS, and we compared them with patients who did not experience C-IRIS or die. We observed that fatal C-IRIS was associated with the upregulation of interferon type 1 and STAT pathways before ART initiation. At the time of C-IRIS, upregulation of transcripts involved in innate immunity (inflammasome and toll-like receptor signaling) was observed in C-IRIS survivors, and many of these same transcripts were upregulated to even higher levels in those who died from C-IRIS. Patients who died but did not have C-IRIS had increased expression of transcripts encoding components of pro-inflammatory pathways associated with oxidative stress and tissue damage. These same pathways were also upregulated at the time of fatal C-IRIS events. These data demonstrated that patients with CM who died or developed fatal C-IRIS had distinct transcriptomic signatures that may have diagnostic and prognostic values.

## Methods

### Sample collection and RNA isolation

This study used deidentified pre-existing samples obtained from the COAT trial performed in Kampala, Uganda. The COAT trial was approved by the University of Minnesota IRB (# 0810M49622), Uganda National Council for Science and Technology, and local Ethics Committees in Kampala, Uganda from Mulago Hospital and Makerere University. These approvals included consents for sample collection, storage, and testing for the studies performed in this manuscript. For more information, and Ethics and Consent to Participate, visit https://clinicaltrials.gov/ct2/show/NCT01075152.

**Fig. 1:**
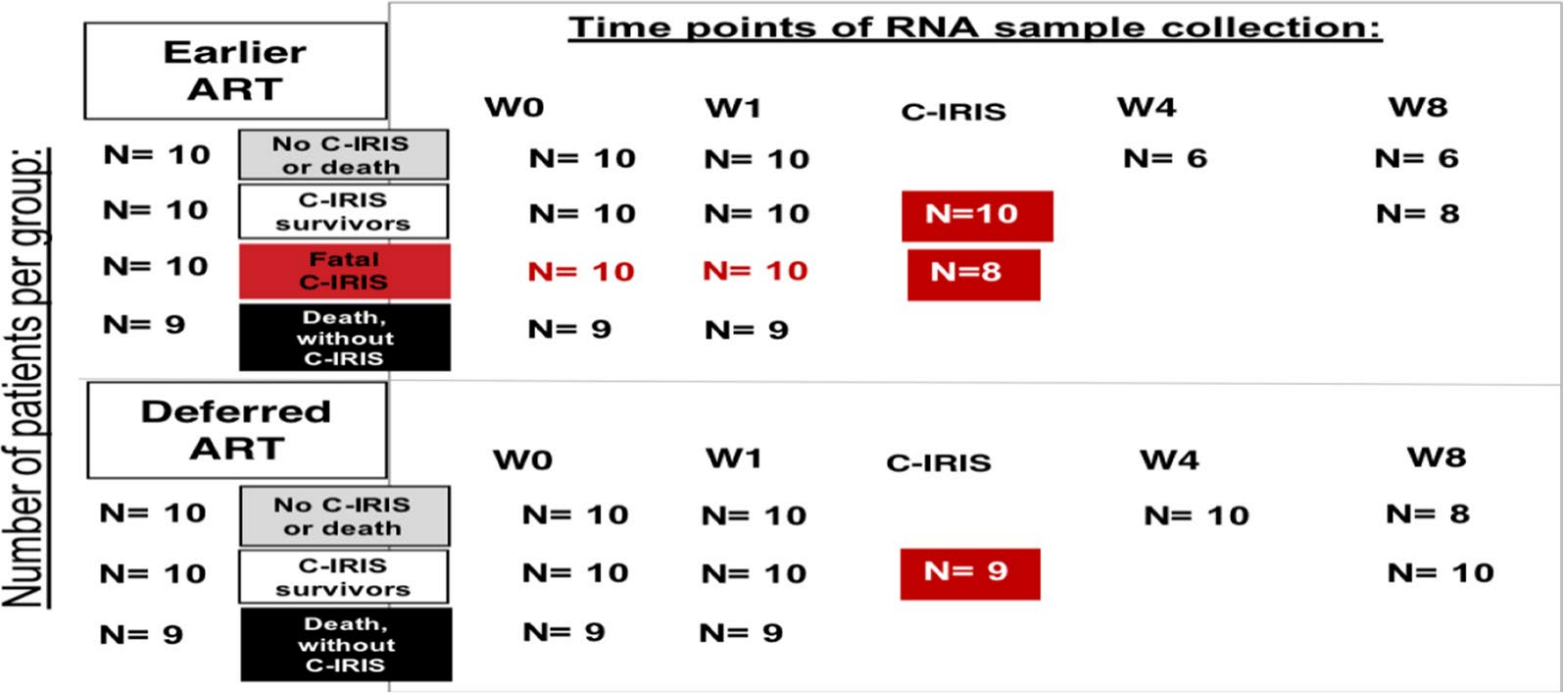
Sample collection and timeline. Blood samples were collected prospectively from HIV-infected Ugandans with CM who were enrolled in the COAT Trial. After COAT study completion, samples from 68 patients were chosen for transcriptome analyses. Of these patients, 39 had been randomized to the earlier ART arm of the COAT Trial, and 29 had been randomized to the deferred ART arm. Ten subjects from each arm did not develop C-IRIS or death (No C-IRIS or Death), 10 subjects in each arm had C-IRIS and survived (C-IRIS Survivors), and 9 subjects in each arm died without C-IRIS (Death without C-IRIS). Ten subjects from the earlier ART arm had C-IRIS and died (Fatal C-IRIS). At the far left of the figure, the number of patients in each of these groups is shown. Blood was collected at randomization immediately prior to ART initiation (W0), one week after ART initiation (W1), at week 4 (W4) and week 8 (W8) after ART initiation, and at the time of C-IRIS events (C-IRIS). The number of samples collected and analyzed at each of these time points for each group of patients is shown

During the trial period, 115 HIV-infected patients with CM were randomized approximately one week post diagnosis to early versus differed ART and were then followed up for 26 weeks [6]. The criteria for C-IRIS diagnosis have previously been published [6]. Deidentified sample processing and analyses were performed at the University of Minnesota. Whole blood (2.5 mL) collected into PAXgene tubes (QIAGEN Inc.) from 68 subjects was assessed by next-generation RNA sequencing. These whole blood samples were collected at the time of ART initiation (week 0), at weeks 1, 4, and 8 after ART commencement and at the time of C-IRIS events (Fig. 1). We categorized patients into 2 groups based on their randomization in the COAT trial: earlier ART initiation arm and deferred ART initiation arm. Patients in each arm were then placed into groups based on their clinical course and outcomes: (1) No C-IRIS or Death Group; (2) C-IRIS Survivor Group; (3) Fatal C-IRIS Group; and (4) Death without C-IRIS Group. Cases of death or C-IRIS occurred within the first month of ART (see Fig. 1).

### Next-generation RNA sequencing

Whole blood was drawn from patients directly into PAXgene RNA tubes (QIAGEN Inc.). These tubes were frozen and shipped to the University of Minnesota. We used the “whole blood PAXgene Blood RNA kit” to extract the ribonucleic acid (RNA) from blood samples (QIAGEN Inc.) according to the manufacturer’s protocol. One microgram of total RNA was measured by RiboGreen RNA Quantification kit (Invitrogen Inc. USA). One microgram was submitted to the University of Minnesota Biomedical Genomics facility for quality controls, assessed by Agilent 2100Bioanalyzer (Agilent Technologies Inc. USA). The Clontech StrandedRNA Pico Mammalian kit was used for library creation. Paired-end (2 × 125 bp or x50bp) sequencing was done on a HiSeq2500 instrument, for 125 cycles, using v4 chemistry (Illumina Inc. USA). The fastq files from this study are deposited in the National Center for Biotechnology Information’s Gene Expression Omnibus and are accessible through GEO Series, ticket number is GSE162914.

### Bioinformatical analysis of gene expression

Fastq files were processed using the Minnesota Supercomputing Institute Unix interface: the qualities of raw reads were assessed and trimmed (in Trimmomatic, http://www.usadellab.org/), if necessary. The alignment and mapping of sequencing reads to a reference genome (GRCh38, https://www.ncbi.nlm.nih.gov/assembly/GCF_000001405.39) was performed with HISAT2 (hierarchical indexing for spliced alignment of transcripts) [8]. Transcript expression was normalized to FPKM (Fragments Per Kilobase of transcript per Million mapped reads) and quantified using Cuffquant (Cufflinks workflow, https://www.msi.umn.edu/sw/cufflinks).

The identification visualization of transcripts that were differentially expressed was done in JMP14.2 Pro (SAS Institute, USA) and Partek® Genomics Suite version 7 (USA). The principal component analysis (PCA) was used to visualize specimen-specific gene expression, as PC correlations between groups (survived vs. died) and subgroups (week 0 versus weeks 1, 4, 8, see Fig. 1). The list of differentially expressed genes was obtained from the analysis of variance with restricted maximum likelihood (ANOVA-REML). Significantly changed probe intensities were filtered based on a minimum fold change threshold of 2 for up- or downregulation (Additional file 3: Table S3). Values of *P* were corrected for multiple testing using the Benjamini–Hochberg false discovery rate.

The enrichment of functional gene categories/pathways was performed in Ingenuity Pathway Analysis (IPA) statistical software. The pathways in IPA refer to a group of functionally or structurally related genes (with known function) that jointly form a network. The IPA workflow comprised core, functional, and canonical pathway analyses and was used as a reference data set. Both direct and indirect molecular relationships were included in the analysis settings, and the significance of relationships between 2200 immune gene transcripts was indicated with z-score and Fisher’s exact test *P* values < 0.05, with false Benjamini–Hochberg discovery rate (FDR) correction.

### Biomarkers search

For identification of biomarkers associated with C-IRIS and Death, the nonlinear iterative partial least squares (NI PLS) algorithm with leave-one-out cross-validation method (available in JMP14.2 Pro (SAS Institute, USA) was used, which was carried out on 2200 transcripts that are part of immune pathways. Variable Importance in the Projection (VIP) scores were extracted to reveal relations between predictors and outcome and plotted against the coefficient of regression. Predictors and responses were scaled to have a mean of 0 and a standard deviation of 1 (by dividing each column by its standard deviation). To consider the transcript as a potential biomarker, the filters were set as such: the VIP score was > 0.8 and the regression correlation coefficient was > 0.2 in at least 1 of the studied subgroups. Model coefficients with their respective ranked VIP contributions are presented in Additional file 4: Table S4. The PLS algorithm is available in Additional file 5: Table S5.

## Results

### Gene expression patterns differed among patients who developed C-IRIS, survived, or died

Our goal was to identify gene expression signatures in peripheral blood associated with the development of death or fatal C-IRIS among patients with CM. We performed next-generation RNA sequencing of samples from 68 HIV-infected subjects with CM who participated in the COAT Trial. Of these, 39 subjects had been randomized to initiate ART within two weeks (earlier ART arm) and 29 subjects had been randomized to initiate ART after 5 weeks (deferred arm) [6]. All subjects received induction anti-fungal therapy with Amphotericin B and fluconazole, followed by consolidation and maintenance therapy with fluconazole as described previously [6]. Of the 68 subjects, 10 subjects in each arm did not develop C-IRIS or death (Control Group), 10 subjects in each arm had C-IRIS and survived (C-IRIS Survivor Group), and 9 subjects in each arm died without C-IRIS (Death without C-IRIS Group). Ten subjects from the earlier ART arm had C-IRIS and died (Fatal C-IRIS Group; see Fig. 1). Cases of C-IRIS were classified based on predefined criteria [5]. All cases of death or C-IRIS occurred within the first month of ART treatment. Peripheral blood was collected before ART initiation (week 0), at weeks 1, 4, and 8 on ART, and at the time of C-IRIS (Fig. 1). Groups had no significant differences in age, sex, pre-ART HIV viral load, or CD4 + T cell count (see Additional file 1: Table S1). The serum cryptococcal antigen titers, as measured by LFA (lateral flow immunoassay), were significantly higher in the C-IRIS Survivor group, as compared to control groups, or patients who died in the earlier ART study arm with or without C-IRIS (*p < 0.05).

**Fig. 2:**
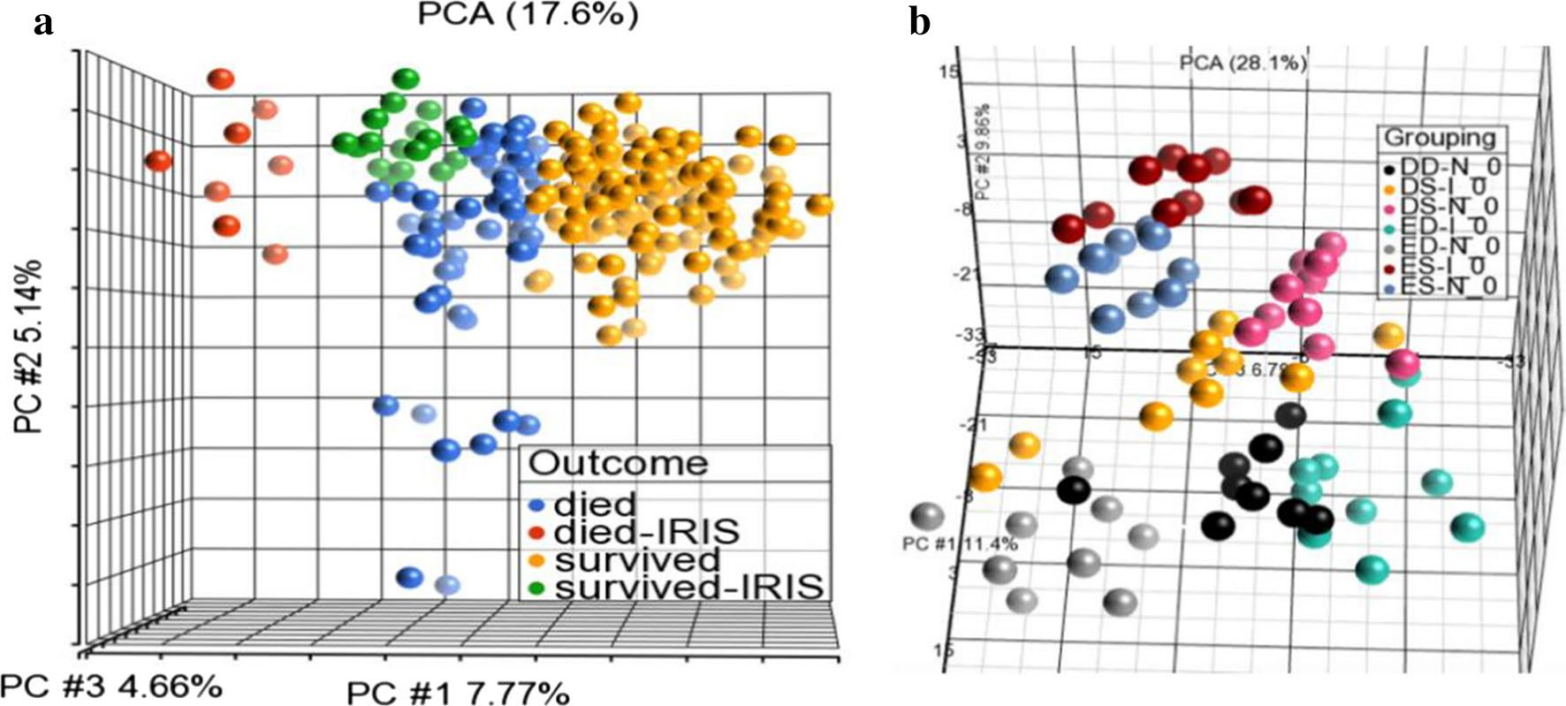
Gene expression patterns distinguish Death, Survival, and C-IRIS. **a** Principal component analysis was used to separate gene expression in 211 individual samples from the 68 subjects. 3-D principal components (PC#) accounted for 17.6% total variance in gene expression (PC#1 7.77%, PC#2 5.14%, PC#3 4.66%, respectively). This PCA was performed on 26,380 expressed RNAs. The outcome box within the plot outlines the color assignments. Samples collected at the time of C-IRIS events are colored as red (died-IRIS = Fatal C-IRIS Group) and green (survived-IRIS = samples from C-IRIS Survivor Group). Samples shown in yellow are from patients who survived (excluding samples collected at the time of C-IRIS events). Samples shown in blue are from those who died (excluding samples collected at the time of C-IRIS events). For the simplicity of representation, the longitudinally assessed time points within each group are not colored differently. **b** PCA subgroup analysis at baseline (at randomization, week 0). This PCA was performed on 2200 expressed RNAs that encode proteins with immune/inflammatory functions. The legend in the plot outlines the color assignment: samples were color-coded by the 7 study groups as shown in the legend box within the figure: DD-N = Deferred arm, Death without C-IRIS Group (black); DS-I = Deferred arm, C-IRIS Survivor Group (yellow); DS-N = Deferred arm, No C-IRIS or Death Group (pink); ED-I = Earlier ART arm, Fatal C-IRIS Group (green); ED-N = Earlier ART arm, Death without C-IRIS Group (grey); ES-I = Earlier ART arm, C-IRIS Survivor Group (maroon); and ES-N = Earlier ART arm, No C-IRIS or Death Group (blue)

We hypothesized that patients with CM would show distinct gene expression signatures in the blood that could distinguish between the four groups: (1) No C-IRIS or Death (control), (2) C-IRIS Survivor Group, (3) Fatal C-IRIS Group, and (4) Death without C-IRIS Group. We applied a principal component analysis (PCA), to visualize the differences in gene expression levels in the entire dataset (26,398 expressed RNAs for each sample) (Fig. 2a). The principal component analysis was used to separate gene expression in 211 individual samples from the 68 subjects. The results separated the samples into four clusters: samples from patients who survived (excluding samples collected at the time of C-IRIS events, yellow dots), samples from those who died (excluding samples collected at the time of C-IRIS events, blue dots), and samples collected at C-IRIS events (red, died; green, survived). As shown in Fig. 2a, samples collected from the C-IRIS Survivor Group at the time of C-IRIS events clustered separately from samples collected from the Fatal C-IRIS Group at the time of fatal C-IRIS events.

We performed additional PCA analysis in order to cluster samples based on the expression of significant immune transcripts at week 0 (pre-ART), from all patient groups (Fig. 2b). The list of all expressed transcripts was truncated to 2200 immune/inflammatory transcripts, and the top 3 principal components were extracted. The immune gene expression at week 0 showed a distinct separation comparing patients who later died of C-IRIS from those who survived (shown as PCA#1, black, grey, and green dots, in Fig. 2b). These data show that gene expression in those who died could already be distinguished from those who survived at the time of ART initiation (week 0), suggesting that gene expression signatures could potentially be used as prognostic markers to define risk for death. These results prompted us to perform further data mining to reveal biological information about transcripts that may contribute to fatal events.

### At week 0 transcriptome profiles differed between surviving patients who did or did not develop C-IRIS

Transcript expression was further compared between groups by ANOVA-REML, using criteria described in the methods. Comparing gene expression at week 0 in surviving patients who did not develop C-IRIS (Control Group) to those who developed C-IRIS (C-IRIS Survivor Group), C-IRIS survivors exhibited lower baseline expression of transcripts associated with antiviral responses, including interferon-induced protein family members, interferon-induced proteins with tetratricopeptide repeats, and oligoadenylate synthetases (IFIs, IFITs, ISGs, and OASs respectively) (Additional file 6: Figure S1a). This confirmed our previously published observation that lower expression of genes encoding interferon type 1- inducible transcripts at the time of CM diagnosis are associated with subsequent nonfatal C-IRIS events [9]. Additionally, we identified elevated expression of transcripts encoding markers of complement components (C1QA, C1QB, C1QC, CFD), HLA-DRB (1 and 5), and IL12 pathways in the C-IRIS Survivor Group at week 0, in comparison to the Control Group (*p* < 0.05) (Additional file 6: Figure S1b). These complement components, HLA-DRs and IL12 play important roles in antigen presentation, which suggests that prior to ART commencement, monocytes and antigen-presenting cells are already activated in C-IRIS survivors. These results suggest that we can use gene expression biomarkers to identify patients at risk for the development of C-IRIS prior to their initiation of ART [9].

### At week 0 transcriptome profiles differed between patients who survived or died

We compared gene expression at week 0 in ten patients from the earlier ART arm who subsequently died from C-IRIS (Fatal C-IRIS Group) to week 0 control patients (No C-IRIS or Death Group) in both study arms. Interestingly, the patients who died from C-IRIS did not exhibit significant deficiency in the expression of interferon-response pathway genes, as compared to controls (the ANOVA results are provided in Additional file 3: Table S3). These results were different from the result described above where patients with C-IRIS who survived had lower expression of these genes. Using Ingenuity Pathway Analysis (IPA) software we combined transcript expression measurements into canonical pathways to identify which pathways act as markers in predicting or explaining fatal outcomes.

**Fig. 3:**
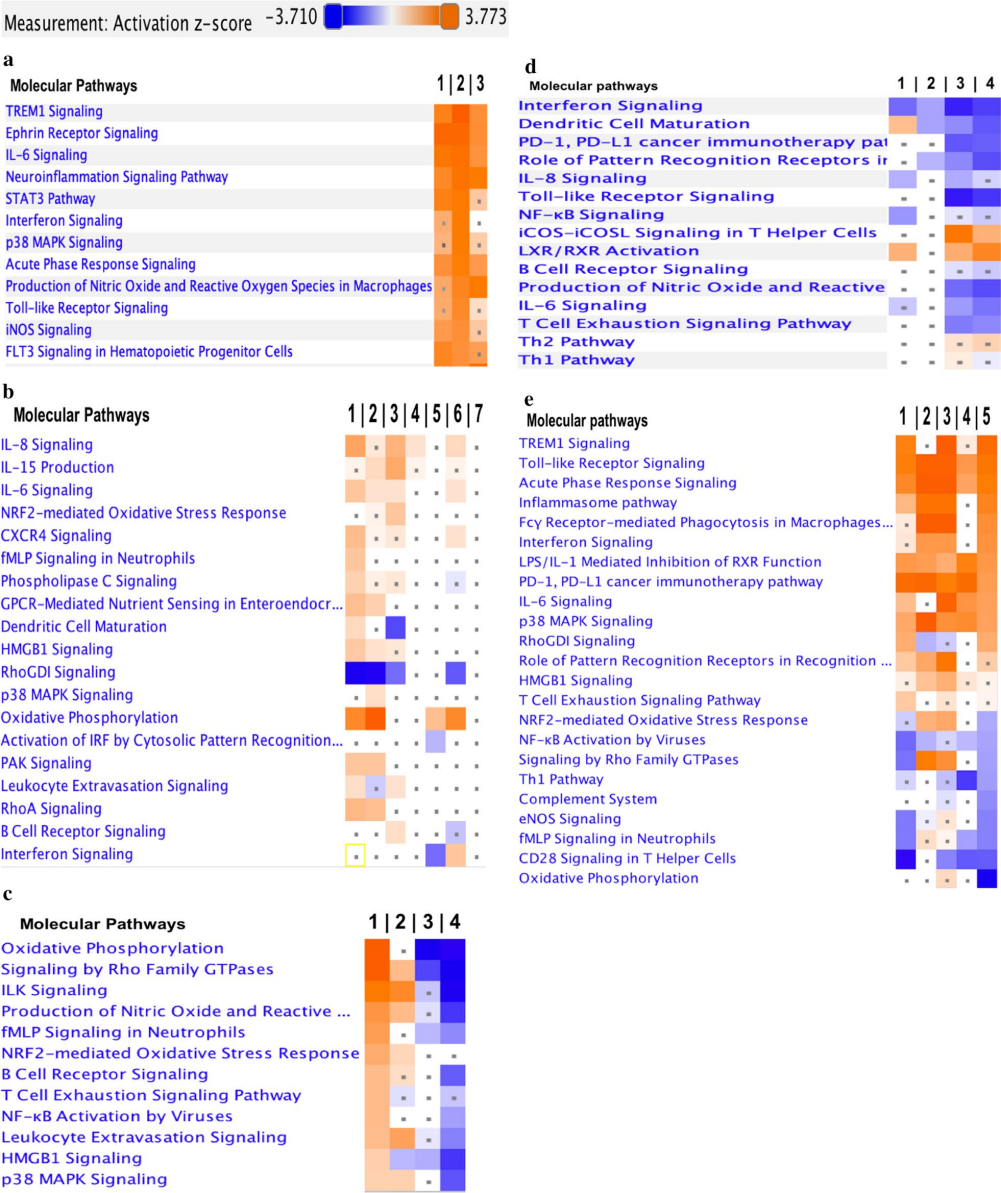
Comparisons of the expression of immune genes and immune/inflammatory pathways between groups. **a–e** Biofunction heatmap illustrations of pathway-level enrichment based on ANOVA analysis are shown. IPA software was used to visualize differences in enrichment of transcripts that are involved in canonical pathways. The heatmaps display top up- and down-regulated pathways. The color of heatmap squares represents the activity of biofunctions. The z-scores represent the significance of fold changes in expressions and colored as activation z-score > 2 (orange squares), repression z-score < -2 (blue), and insignificant z-score (white squares). Dots mark the squares which did not pass Benjamini–Hochberg restrictions for changes in gene expression within the canonical pathway. vs., versus. The ranges of z-scores identified in this analysis are between − 3.7 and + 3.7. **a.** Upregulation of immune pathways in the Fatal C-IRIS Group at week 0. 1. **Column 1**. Fatal C-IRIS Group versus No C-IRIS or Death Group (control) from both study arms. 2. Fatal C-IRIS Group versus C-IRIS Survivor Group. 3. Fatal C-IRIS Group versus Death without C-IRIS Group. **b**. Changes in pathway expression at one week after ART initiation as compared to corresponding week 0. 1. Death without C-IRIS Group, week 1 versus week 0 (earlier ART arm). **Column 2**. Death without C-IRIS Group, week 1 versus week 0 (deferred ART arm). **Column 3**. Fatal C-IRIS group, week 1 versus week 0 (earlier ART arm). **Column 4**. No C-IRIS or death Group week 1 verus week 0 (earlier ART arm). **Column 5**. No C-IRIS or death Group week 1 versus week 0 (deferred ART arm). **Column 6**. C-IRIS Survivors Group week 1 versus week 0 (earlier ART arm). **Column 7**. C-IRIS Survivors Group week 1 versus week 0 (deferred ART arm). **c** Pathway expression comparisons between Groups, at 1 week post-ART (earlier and deferred arms combined). **Column 1**. Death without C-IRIS Groups versus No C-IRIS or Death Groups (controls). **Column 2**. Fatal C-IRIS Groups versus No C-IRIS or Death Groups (controls). **Column 3**. Fatal C-IRIS Group versus Death without C-IRIS Groups. **Column 4**. C-IRIS Survivor Groups versus Death without C-IRIS Groups. **d** Changes in pathway expression at 8 weeks after ART initiation. **Column 1**. Earlier ART arm: No C-IRIS or Death Group (control), week 8 versus No C-IRIS or Death Group (control), week 0 and 4 combined. **Column 2**. Deferred ART arm: No C-IRIS or Death Group (control), week 8 versus No C-IRIS or Death Group (control), week 0 and 4 combined. **Column 3**. Earlier ART arm: C-IRIS Survivor Group, week 8 versus C-IRIS Survivor Group at the time of C-IRIS event. **Column 4**. Deferred ART arm: C-IRIS Survivors Group, week 8 versus C-IRIS survivor Group at time of C-IRIS event. **e** Immune pathways that were up-down-regulated at the time of C-IRIS events in the earlier arm. **Column 1**. Fatal C-IRIS Group (event) versus Fatal C-IRIS Group, 1 week (most proximal time point to the event). **Column 2**. Fatal C-IRIS Group (event) versus No C-IRIS or Death Group (control) at 1 week post-ART. **Column 3**. Fatal C-IRIS Group (event) versus No C-IRIS or Death Group at week 4 post-ART. **Column 4**. Fatal C-IRIS Group (event) versus C-IRIS Survivor Groups (event). **Column 5**. Fatal C-IRIS Group (event) versus Death without C-IRIS Group at week 1.

We ran a comparison analysis of canonical pathways and built heatmaps of the top up and down-regulated pathways. We identified twelve inflammatory immune pathways that were upregulated at week 0 in the Fatal C-IRIS Group as compared to the three other groups from either study arm: No C-IRIS or Death Group (Control), C-IRIS Survivor Group, and Death without C-IRIS Group. Since the results were almost identical, we combined groups from both study arms together (see Fig. 3a, columns 1, 2, 3, respectively). These 12 pathways included cytokines and other pro-inflammatory molecules such as CXCL1, CXCR1, ICAM1, IL6, IL8, IL11, interferons, and components of MAPK signaling (MAPK14, NFKBIA, IL1 receptors). No significantly downregulated pathways were identified, suggesting that patients who died due to C-IRIS, were not immunocompromised to a higher degree than patients from other groups, since they were not deficient in immune gene expression.

Notably, patients from the Death without C-IRIS Group did not show any statistically significant upregulation of immune gene expression, except for type 1 interferon signaling, when compared to the No C-IRIS or Death (Control), or the C-IRIS Survivor Group (week 0). The Death without C-IRIS Group, however, exhibited a trend towards a downregulated expression of various HLA-, KLRC-, Th1- and Th2- pathways, but upregulation of PD1/PDL1, COX, and FCGR- transcripts at the time of ART initiation, which may reflect adaptive immune cell exhaustion and impaired phagocyte function (z scores < 1). The fold change in expression values for these transcripts are presented in Additional file 2: Table S2.

Together, these results suggest that gene expression at week 0 differed between the Fatal C-IRIS Group, the Death without C-IRIS Group, and the survivors (Fig. 3a).

### Changes in gene expression after ART initiation differed among those who died or survived in the earlier and deferred ART initiation arms

Using the same pathway-based biomarker discovery approach, we assessed changes in pathway expression over time among patients in the earlier and deferred ART initiation arms. At one week of observation on ART, studied groups exhibited upregulation of similar components of pro-inflammatory pathways, when compared to week 0 within each corresponding arm (Fig. 3b). Patients in the Death without C-IRIS Group, whether they were in the earlier or deferred ART arms, showed a distinct shift toward upregulation of transcripts involved in oxidative phosphorylation, high mobility group box 1 proteins (HMGB1), pro-inflammatory cytokine signaling (IL6, 8, 15), and Rho/GTPase pathways (Fig. 3b, columns 1, 2). A similar trend was seen in the Fatal C-IRIS Group at one week after ART initiation (Fig. 3b, column 3). Interestingly, Ras homologous Guanosine diphosphate (GDP)-dissociation inhibitors, RhoGDIs, which negatively regulate Rho family GTPases, were downregulated in all Death Groups (with or without C-IRIS), at 1 week after ART initiation. Thus, the No C-IRIS or Death Groups and the Death without C-IRIS Groups showed distinct gene expression changes regardless of the timing of ART initiation.

Control groups (in earlier and deferred ART arms) did not exhibit significant changes at week 1 compared to week 0 in these proinflammatory pathways, (Fig. 3b, column 4, 5). At week 1, patients in the C-IRIS Survivor Group from the earlier ART arm exhibited more dramatic upregulation of proinflammatory gene expression as compared to those in the C-IRIS Survivor Group who were in the deferred ART arm (Fig. 3b, column 6, 7).

Comparison between samples, collected at week one post-ART showed that the Death without C-IRIS Group exhibited the most pronounced changes in gene expression in earlier and deferred ART arms (Fig. 3c). Numerous transcripts within granulocyte- activation pathways, such as N-formyl-Met-Leu-Phe (fMLP pathway), HMGB1, and Rho family GTPases (Rho GTPases), were upregulated in the Death without C-IRIS Group, as compared to the controls. The oxidative phosphorylation pathway was the most overexpressed, followed by Rho family GTPase pathways and stress kinase pathways (Fig. 3c, column 1). The Fatal C-IRIS Group exhibited similar gene expression changes, when compared to controls (Fig. 3c, column 2). However, when compared to Death without C-IRIS Groups (earlier and deferred ART arms combined), the oxidative stress and neutrophil involvement signatures were not present at week 1 of observation (Fig. 3c, column 3). In comparison, C-IRIS survivors showed significantly lower expression of 10 out of the 12 pathways presented in Fig. 3 (see column 4) when compared to Death without C-IRIS Groups. These results indicate that the transcriptomic signature at the time of near death, in the Death without C-IRIS Group, is different than in those of comparators at the closest time points of observation.

### Immune recovery over 8 weeks on ART in control patients without C-IRIS or death

Longitudinal analysis of immune reconstitution during 8 weeks on ART in the No C-IRIS or Death (control) Groups, who had a favorable clinical recovery, revealed downregulation of interferon signaling and NF-kappa B signaling pathways, but upregulation of phagocyte maturation pathways (Fig. 3d, columns 1, 2). This course of immune recovery on ART was similar to what we previously demonstrated in advanced stage HIV-infected patients without opportunistic infections after initiation of ART [10]. We also assessed changes in gene expression in the C-IRIS Survivor Group in the period following C-IRIS events. The C-IRIS Survivors Group exhibited downregulation of eight signaling pathways at week 8 on ART (most proximal post-C-IRIS time point) when compared to the gene expression at C-IRIS events (Fig. 3d, columns 3, 4). For example, Toll-like receptor signaling, pro-inflammatory IL6, IL8, and T cell exhaustion pathways were downregulated, which may represent a sign of recovery from chronic antigen exposure and a prolonged stage of chronic inflammation [10, 11]. Additionally, transcripts involved in Th1 and Th2 pathways showed a trend towards upregulation, but this trend did not pass the threshold of a significant z-score (Fig. 3d, columns 3, 4). Perhaps, assessment of longer time points of evaluation, such as 12 and 26 weeks post-ART, would show a more significant recovery in the expression of T cell pathways.

### Dysregulated immune gene expression at the time of fatal C-IRIS

The major goal of this study was to identify altered gene expression at fatal C-IRIS events in order to better understand the molecular pathology of fatal C-IRIS. Analysis of changed gene expression at the time of fatal C-IRIS revealed many significantly upregulated acute phase response and oxidative stress pathways (e.g., IL1 and IL6, TLR, HMGB1, NRF2-mediated, NFkB, p38-MAPK), when compared to the most proximal longitudinal time point (W1) within the fatal C-IRIS group (Fig. 3e column 1), or the No C-IRIS or Death (control) Group at week 1 (Fig. 3e column 2).

Since fatal C-IRIS cases occurred within 4 weeks of ART initiation and only in the earlier ART arm, we compared gene expression in the Fatal C-IRIS Group to the Control Group collected at week 4 post-ART in the earlier ART arm. The Fatal C-IRIS Group showed significant upregulation of more than a dozen inflammatory pathways (Fig. 3e column 3). This result implies that the acute inflammation observed in the Fatal C-IRIS Group was not seen in the Control Group, perhaps due to favorable immune reconstitution with antifungal treatment and ART.

Transcripts involved in acute phase response pathways, Toll-like receptor signaling, IL1, IL 6, and p38 MAPK were upregulated in the fatal C-IRIS group as compared to C-IRIS Survivor Groups, during C-IRIS events (Fig. 3e, column 4). The lower expression of transcripts involved in Th1-, CD28-, and other T cell-related pathways, but upregulation of transcripts involved in T cell exhaustion signaling pathways (PD1/PDL1), was observed during fatal C-IRIS events when compared to C-IRIS events in the C-IRIS Survivor Group (Fig. 3e, column 4).

In comparison to the Death without C-IRIS Group, the Fatal C-IRIS Group showed upregulation of the same acute phase response pathways and similar expression of HMGB1 and T cell exhaustion signaling, but downregulation of complement and oxidative stress pathways (eNOS, NRF2-mediated, Rho family GTPases, fMLP, etc.) (Fig. 3e, column 5). These results indicate a divergence between activation pathways that are associated with death without C-IRIS and death due to C-IRIS.

### Transcriptomic biomarkers predict fatal C-IRIS or death

To identify transcripts that may be predictive biomarkers of fatal C-IRIS events, we used probability modeling based on a partial least squares (PLS) computational algorithm, as described in the methods. PLS analysis was performed for samples from the C-IRIS Survivor Group and Fatal C-IRIS Group, with comparisons to the rest of the groups, including all time points within each group. The models were carried out on the list of 2200 expressed transcripts that are significantly enriched in immune pathways.

**Fig. 4:**
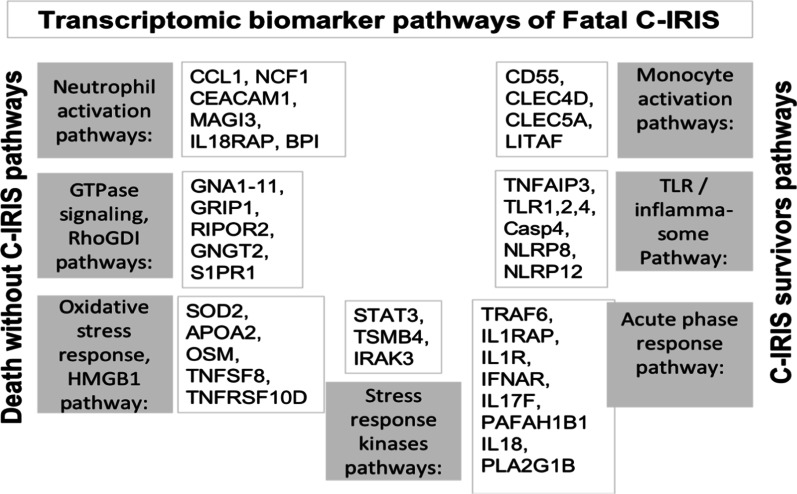
Transcriptional biomarkers and biomarker pathways that drive fatal inflammation during C-IRIS events. These biomarker transcripts were identified using a partial least square algorithm. The pathways that play a role in fatal outcome are depicted in grey boxes. The transcripts that are unique to the Fatal C-IRIS Group are depicted in clear boxes. The pathways shared with the Death without C-IRIS Group are depicted on the left; the pathways shared with the C-IRIS Survivor Group are depicted on the right*.* Pathways shared by all three groups are depicted on the bottom. Our model suggests that the overactivated innate immune system, driven by monocytes via inflammasome/TLR2,4 pathways, drives C-IRIS events (right). Excessive neutrophil activation via fMLP, Rho family GTPases, HMGB1 and inactive RhoGDI pathways results in cellular and tissue damage promoting the pathogenesis of fatal events (left)

The model revealed that most of the top-ranked biomarkers were distinct and specific for either fatal or non-fatal C-IRIS events, and the rest were similar between these groups. For example, differential expression of transcripts encoding IL15, IL31, integrins (ITGA7, ITGB2), and SIGLECs (sialic acid binding immunoglobulin—like lectins) were ranked as highest importance for nonfatal C-IRIS events (C-IRIS Survivor Groups), but not for fatal C-IRIS events (see Table 1). Conversely, p38 MAPK signaling, IL1R, IL18R, TLR1,2,4, NLRP8,12, and transcripts encoding CLECs (C-type lectins), ranked as higher importance for fatal C-IRIS (Fatal C-IRIS Group), but not for C-IRIS survivors (Fig. 4).

**Table 1:** The comparison of transcriptomic biomarkers between fatal and nonfatal C-IRIS

Gene	Fatal C-IRIS group		C-IRIS survivor group, earlier arm		C-IRIS survivor group, deferred arm	
Symbol	R for VIP	VIP rank	R for VIP	VIP rank	R for VIP	VIP rank
LIMK1	− 0.0116	− − − − −	0.0054	+ + + +	0.015	+ + + +
ALOX5	− 0.0101	− − − −	0.0026	+ +	0.0102	+ + +
CTSC	− 0.0087	− − − −	0.0056	+ + + +	0.0137	+ + + +
IL17REL	− 0.0092	− − − −	0.0033	+ +	0.0074	+ +
TLR10	− 0.0089	− − − −	0.0027	+ +	0.012	+ + +
C3AR1	− 0.0086	− − −	0.0058	+ + + +	0.012	+ + +
CTSD	− 0.0064	− − −	0.0038	+ + +	0.0054	+ +
CTSK	− 0.0086	− − −	0.0045	+ + +	0.0103	+ + +
DEFB115	− 0.007	− − −	0.0057	+ + + +	0.0122	+ + +
IL17D	− 0.0068	− − −	0.0036	+ +	0.0111	+ + +
ITGA7	− 0.0077	− − −	0.0058	+ + + +	0.0086	+ +
ITGB2	− 0.0067	− − −	0.0052	+ + +	0.0086	+ +
JAG1	− 0.0085	− − −	0.0034	+ +	0.011	+ + +
SIGLEC10	− 0.0074	− − −	0.0065	+ + + +	0.0118	+ + +
ALPL	− 0.0044	− −	0.0063	+ + + +	0.0103	+ + +
CASP5	− 0.0042	− −	0.0045	+ + +	0.0121	+ + +
DEFA5	− 0.0044	− −	0.0047	+ + +	0.0047	+
ITGAX	− 0.0061	− −	0.0069	+ + + + +	0.0106	+ + +
NOD2	− 0.0043	− −	0.0043	+ + +	0.0054	+ +
NOTCH2	− 0.0045	− −	0.0048	+ + +	0.0063	+ +
NOX1	− 0.004	− −	0.0048	+ + +	0.0044	+
SIGLEC5	− 0.0044	− −	0.0037	+ + +	0.0084	+ +
SIGLEC7	− 0.0057	− −	0.0047	+ + +	0.0091	+ + +
BEX1	− 0.0033	−	0.0027	+ +	0.0026	+
CASP2	− 0.0034	−	0.0038	+ + +	0.0063	+ +
ITGA5	− 0.0034	−	0.0056	+ + + +	0.0043	+
SIRPA	− 0.0018	−	0.0038	+ + +	0.007	+ +
IL1RAP	0.0146	+ + + + + +	− 0.002	−	− 0.0071	− −
MMP20	0.0117	+ + + + +	− 0.0037	− −	− 0.0086	− −
ALOX5AP	0.0087	+ + + +	− 0.0035	− −	− 0.0073	− −
CCL1	0.0103	+ + + +	− 0.004	− − −	− 0.0079	− −
CD55	0.0089	+ + + +	− 0.0005		− 0.0041	−
CLEC4D	0.0102	+ + + +	− 0.0027	− −	− 0.0055	− −
CLEC4E	0.0106	+ + + +	− 0.0049	− − −	− 0.0068	− −
CLEC5A	0.0091	+ + + +	− 0.0032	− −	− 0.004	−
F2RL2	0.0088	+ + + +	− 0.0011	−	− 0.0058	− −
FADD	0.0102	+ + + +	− 0.0037	− − −	− 0.0071	− −
FAM65B	0.0091	+ + + +	− 0.0006	−	− 0.0029	−
IFNAR1	0.0094	+ + + +	− 0.0018	−	− 0.0037	−
IL18R1	0.0106	+ + + +	− 0.0018	−	− 0.0032	−
IL18RAP	0.0101	+ + + +	− 0.0016	−	− 0.0049	−
IL1R1	0.01	+ + + +	− 0.0019	−	− 0.0052	−
IRAK3	0.0088	+ + + +	− 0.0007	−	− 0.0042	−
KLKP1	0.0102	+ + + +	− 0.0038	− − −	− 0.0064	− −
LILRB5	0.0106	+ + + +	− 0.0013	−	− 0.006	− −
TLR4	0.0096	+ + + +	− 0.003	− −	− 0.0046	−
CASP4	0.0065	+ + +	− 0.003	− −	− 0.0021	−
CD59	0.0082	+ + +	− 0.0003		− 0.0003	
CLEC2L	0.0063	+ + +	− 0.0044	− − −	− 0.0102	− − −
CXCR1	0.0065	+ + +	− 0.0011	−	− 0.0004	
EXOSC4	0.0081	+ + +	− 0.0018	−	− 0.002	−
FAM188A	0.0076	+ + +	− 0.0031	− −	− 0.0049	−
IFNGR1	0.0066	+ + +	− 0.0002		− 0.0017	
IL17F	0.0086	+ + +	− 0.0028	− −	− 0.0043	−
IL1R2	0.0084	+ + +	− 0.0006		− 0.0054	− −
KLF2	0.0072	+ + +	0.0018	+	− 0.0025	−
KLF6	0.0064	+ + +	− 0.0008	−	− 0.0022	−
MMP8	0.0068	+ + +	− 0.0006		− 0.006	− −
CLEC4F	0.0039	+ +	− 0.0036	− −	− 0.0065	− −
IFRD1	0.005	+ +	0.0009	+	− 0.0028	−
IL1RL1	0.0048	+ +	− 0.0032	− −	− 0.0045	−
IL1RN	0.0049	+ +	− 0.0028	− −	− 0.0016	
MMP9	0.0057	+ +	0.0004		− 0.0042	−
MOSPD2	0.0044	+ +	0.0001		− 0.0019	−
TLR2	0.0059	+ +	− 0.0007		− 0.0006	
HIV_RNA	0.0017	+	− 0.0018	−	− 0.0015	
IL1RL2	0.0032	+	0.0023	+ +	− 0.0011	
SIGLEC9	0.0023	+	0.003	+ +	0.0038	+
IFNAR2	0.0008		0.0025	+ +	0.0019	+
NLRP3	− 0.0009	−	0.0054	+ + + +	0.0079	+ +

Number of “−” is a visual representation for predictive importance strength that the following transcript's expression is negatively associated with occurrence of IRIS events. One “−” is equal 0.7 on VIP scale

Number of “+” is a visual representation for predictive importance strength that the following transcript’s expression is positively associated with occurrence of IRIS events. One “+” is equal 0.7 on VIP scale

*VIP* variable importance in the projection values, *R for VIP* Correlation Coefficient of Least Square Mean for VIP

The PLS model identified the list of novel contributive biomarker transcripts with high importance for the Death without C-IRIS Groups (earlier and deferred ART arms combined), but not for fatal or nonfatal C-IRIS. These included C1QTNF2 and 5, CD207, CD209, CXCL3 and 11, CCL28, MIP, defensins (DEFA6, DEFB107A, DEFB116, DEFB118, etc.) (Additional file 4: Table S4).

Additionally, the PLS model identified the high importance of many interferon-response genes, CLEC4F, NLRP5, CD4, CD1C, CCL21, CCR10, as week 0 predictors of subsequent nonfatal C-IRIS. This model also identified FN1 (fibronectin), LILRB4, SERPINE1, CD80, HLA-DQB2, FERM2 (fermitin 2), RND2, CCDC42 (Rho family GTPases), IL11, IL31, and many other transcripts within several stress response kinases pathways, to be week 0 predictors for subsequent fatal C-IRIS. Conversely, biomarkers such as NLRP6, C3, C5, CTSO, AGTR1, CDH9, etc. were identified as week 0 predictors of death without C-IRIS.

The complete listing of immune biomarkers with a quantitative estimation of the discriminatory power of each predictor transcript provided by utilizing VIP scores and the correlation coefficient (R for VIP) is presented in Additional file 4: Table S4. We were unable to provide pathway comparisons since pathways for many novel top-ranked biomarkers identified by PLS were not available in IPA. Overall, our results suggest that immune and inflammatory biomarkers that we discovered, could be useful for identifying patients with CM who are at risk for dying from C-IRIS.

## Discussion

Despite improved clinical care in patients with advanced HIV-associated CM, overall mortality remains high, highlighting the need for a better molecular understanding of the pathophysiology of fatal outcomes. The biomarkers that we identified in this study provide insight into the pathophysiology of C-IRIS and may be useful for identifying patients with CM who are at the highest risk for death [12]. We identified two gene expression patterns associated with death in patients with CM. The first pattern was seen in patients who developed C-IRIS and died, and the second pattern was seen in patients who died from CM but did not develop C-IRIS.

We have previously reported that HIV-infected patients with CM show a transcriptomic signature of aberrant innate immune activation in the blood during non-fatal C-IRIS events [9]. We showed that C-IRIS survivors exhibited significantly lower expression of transcripts encoding interferon type 2 and antiviral defense proteins prior to ART commencement. Patients who experienced fatal C-IRIS did not show downregulation of interferon-response genes prior to ART initiation, which was one feature that set apart this group from survivors. We suggest that screening of IFN-response gene expression in patients prior to ART initiation could potentially be used to estimate the risk of deadly C-IRIS development [13]. Additionally, we identified numerous molecules within 12 proinflammatory pathways (Fig. 3a), that were upregulated prior to ART commencement in those who developed fatal C-IRIS. Upregulation of these pathways may predispose patients to detrimental clinical sequela after ART initiation, perhaps by promoting the exaggerated inflammatory response associated with innate immune system activation in fatal C-IRIS [14, 15].

The effect of timing of ART initiation in the earlier and deferred ART arms of the COAT trial did not significantly influence proinflammatory gene expression in the control groups (Fig. 3b). The increase of the oxidative stress response pathway was observed in the C-IRIS survivors from the earlier ART arm, as compared to the C-IRIS survivors from the deferred arm. The ART-induced shift in gene expression in the Fatal C-IRIS Group had more similarity with Death without C-IRIS Groups (from both study arms) than with C-IRIS survivors. Activation of pro-inflammatory pathways listed in Fig. 3b, c, suggests that individual fungal antigen burden may play a more important role in driving the fatal outcome. This indicates that assessing the effectiveness of antifungal therapy to achieve fungal clearance before initiation of ART is important to prevent fatal outcomes. Longitudinal analysis of immune recovery on ART in the groups of survivors was similar to previously demonstrated, where downregulation of innate immune proinflammatory pathways was observed after 8 weeks of observation (Fig. 3d) [10]. The reduced expression of these pathways appears to be beneficial for effective immune reconstitution on ART.

In this study, we identified commonly upregulated pathways, between fatal C-IRIS and C-IRIS survivors, such as inflammasome pathway and a part of Toll-like receptor signaling, with the observation that the magnitude of the upregulation of the inflammatory innate immune signature was more pronounced in the fatal C-IRIS group. In comparison to patients who survived C-IRIS events, patients who subsequently died from C-IRIS showed a unique immune signature that included upregulation of many acute phase response signaling pathways (e.g., IL1, IL6, TLR), and PD1/PDL1 T cell exhaustion signaling pathways (Fig. 3e).

We identified several common molecular pathways between the Fatal C-IRIS and Death without C-IRIS groups, such as fMLP, HMGB1, Rho family GTPases, and stress response kinase pathways (Fig. 3e). Although the upregulation of these pathways in the Fatal C-IRIS Group was not as high compared to the Death without C-IRIS Group, upregulated pathways such as TLR- and inflammasome pathways (which are common with those of C-IRIS survivors) perhaps contributed to fatal outcomes during C-IRIS. Cumulatively, these pathways are part of oxidative stress responses and innate immune defenses often described in relation to neuroinflammation, mitochondrial dysfunction and very likely reflect the degree of systemic inflammation [16, 17]. FMLP, RhoGDI (Ras homologous GDP dissociation inhibitors), and Rho family GTPases are essential pathways for proper neutrophil activation and function [18]. They involve activation of multi-subunit enzymatic cascades (e.g., NADPH oxidases), which produce reactive oxygen species (ROS) and reactive nitrogen intermediates [19]. Future investigations into the precise role for neutrophils and fMLP pathway, the Rho family GTPases, and their inhibitors RhoGDI during oxidative stress, in fatal outcomes, may lead to future therapies search that would balance intracellular production of reactive oxygen species [20].

Based on the pathway analyses and predictor screening model results, we composed a map of transcriptional biomarker pathways that appear to drive inflammation during fatal C-IRIS (Fig. 4). This working model included seven predominant immune pathways that are altered during Fatal C-IRIS events, and also in those of survivors and those who died without C-IRIS. These pathways represent an activated innate immune cell signaling in response to both pathogen-specific and endogenous danger signals, which occurs on the background of CD4 + T lymphocytes exhaustion (based on overexpression of PD1/PDL1 pathways, Fig. 3e) [21].

Our proposed molecular pathogenesis model suggests that prior to fatal events, severe and systemic oxidative stress results in cellular necrosis and apoptosis of immune and non-immune cells via pathways depicted in Fig. 4 [22]. Released HMGB1 protein promotes neutrophil recruitment from the bloodstream to brain tissues, by means of inflammatory changes of the endothelium and the sub-endothelium of the blood–brain barrier [23, 24]. HMGB1 is an endogenous alarmin (danger signal molecule) that mediates the release of chemokines, pro-inflammatory cytokines, and lipid mediators [23, 25]. Reactive oxygen intermediates, released by activated granulocytes, set up a damaging environment, accompanied by the loss of endothelial barrier integrity and the trafficking of inflammatory cells across the endothelial barriers [26]. The overproduction of ROS by monocytes and neutrophils in combination with the overactive inflammasome pathway sets up a systemic inflammation that can be fatal to the host.

Our study is limited by relatively small group sizes. Despite this, we observed dramatic differences in gene expression patterns in these study participants with CM that distinguish those who had normal immune reconstitution after ART from those who developed C-IRIS or those who died. A larger study in a different cohort of patients with CM should be performed to validate our results.

## Conclusions

Further work into an understanding of the molecular drivers of death and C-IRIS in patients with CM is needed to develop better diagnostic and prognostic tests and guide novel targeted treatments.

## Supplementary Information

**Additional file 1.** **Table S1.** Baseline (week 0) patient characteristics for each group: means, standard deviations and Lower/upper 95% confidence interval per group. Assessment of differences in age, CD4+ T cell count, pre-ART HIV viral load, Serum CrAg Titers (lateral flow immunoassay), and sex between groups. Wilcoxon rank sum paired test for nonparametric data was used for the comparison between the groups (JMP14.2 Pro, SAS Institute). *The cut-off significance level for all *P* values < 0.05.The pre-ART serum cryptococcal antigen titers (CrAg), as measured by LFA assay, were significantly higher in C-IRIS survivors’ group, as compared to patients who died in earlier ART study arm, with or without C-IRIS.

**Additional file 2.** **Table S2.** Fold change in expression for genes within phagocytes- and T cell pathways, in Death without C-IRIS group at week 0. The comparison performed between Death without C-IRIS groups and No C-IRIS or Death groups (from early and deferred ART arms combined).Benjamini-Hochberg-corrected Z scores that are < 1 for these pathways’ identification, (are equal to ‘not significant for all’). The expression fold changes, and *p*-values are calculated by ANOVA-REML (see methods). Expression *p*-value, with no FDR (no false discovery rate) applied, are shown.

**Additional file 3.** **Table S3.** Gene expression comparisons results: analysis of variance with restricted maximum likelihood (ANOVA-REML). ANOVA multivariate analysis was performed as described in the methods. Group assignment legend: DD-N = Deferred Arm, Death without C-IRIS Group; DS-I = Deferred Arm, C-IRIS Survivor Group; DS-N = Deferred Arm, No C-IRIS or Death Group; ED-I = Earlier Arm, Fatal C-IRIS Group; ED-N = Earlier Arm, Death without C-IRIS Group; ES-I = Earlier Arm C-IRIS Survivor Group; ES-N = Earlier arm, No C-IRIS or Death Group. _0; _1; _4; _8; = 0, 1, 4, 8 weeks after ART, respectively. 4i = at the time of C-IRIS events.

**Additional file 4.** **Table S4.** Biomarkers identified by Partial Least Square model. Partial Least Squares (PLS) regression model coefficients (R) for centered and scaled Various Importance in Projection (VIP) data. This data was analyzed by Groups: earlier and deferred ART arms combined (see the methods section and the text).

**Additional file 5.** **Table S5.** PLS algorithm script. Partial Least Squares (PLS) algorithm script exported from JMP14.2 Pro.

**Additional file 6.** **Figure S1a, S1b.** The fold change expression values for up- and down-regulated transcripts in C-IRIS Survivor Group, as compared to NoC-IRIS or Death Group. **S1a.** The low antiviral gene expressions at the time of antiretroviral therapy (ART) initiation in cryptococcosis-associated immune reconstitution inflammatory syndrome (C-IRIS) group. **S1b.** An example of upregulated transcripts in C-IRIS Survivor Group, as compared to No C-IRIS or Death Group, at week 0.

## Data Availability

The fastq files from this study are deposited in the National Center for Biotechnology Information’s Gene Expression Omnibus (GEO) and are accessible through GEO ticket number GSE162914, at: https://www.ncbi.nlm.nih.gov/geo/query/acc.cgi?acc=GSE162914.

## Abbreviations

IRF: Interferon regulatory factor
IFI: Interferon alpha inducible protein
IFIT: Interferon induced protein with tetratricopeptide repeats
IFNG: Interferon gamma
RSAD2: Radical S-adenosyl methionine domain containing 2
OAS: 2′-5′-Oligoadenylate synthetases
DHX58: DExH-box helicase (LGP2)
DDX58: DExD/H-box helicase 58 (RIG1)
C1QB, C1QC: Components of complement
TREM1: Triggering receptor expressed on myeloid cells 1
FCGR: Fc fragment of IgG receptors
KLRC: Killer cell lectin like receptors
COX: Cytochrome c oxidase subunits
HLA-: Major histocompatibility complex, class I human leucocyte antigens
Rho Family GTPase 2/Rho GTPases: Ras homologous family guanosine triphosphatases
fMLP pathway: N-formyl-Met-Leu-Phe or N-Formylmethionyl-leucyl-phenylalanine
HMGB1: High mobility group box-1
VIP: Variable importance for the projection score
NRF2: Nuclear factor erythroid 2 (NF-E2)—related factor
FN1: Fibronectin1
C1QTNF2: Complement C1q and tumor necrosis factor-related
PAMP: Pathogen-associated molecular patterns
CD207/CLEC4K: C-type lectin (CTL)/C-type lectin-like (CTLD) domain; CD209/CLEC4L
CLEC4F, CLEC: C-type lectin domain family members
CXCL3 and 11: C-X-C Motif Chemokine Ligands
SIGLEC: Sialic acid binding immunoglobulin like lectins
RSAD2: Radical S-adenosyl methionine domain containing 2
RhoGDI: Ras homologous GDP dissociation inhibitors, GDIs
CCL28: C–C Motif Chemokine Ligand
MIP/CCL3L3: C–C motif chemokine ligand 3 like 3
DEFA6, DEFB107A, DEFB116: DEFB118 defensins A/B
NLRP5,6: NLR (NOD-like receptor) family pyrin domain containing 5
CCL21: C–C Motif Chemokine Ligand 21
CCR10: C–C Motif Chemokine Receptor 10
LILRB4: Leukocyte Immunoglobulin Like Receptor B4
SERPINE1: Serpin Family E Member 1
FERM2: Fermitin 2
RND2: Ras Homolog Gene Family Member N
CCDC42: Coiled-coil domain containing 42
C3, C5: Complement components
AGTR1: Angiotensin II receptor type 1
CDH9: Cadherin 9
CTSO: Cathepsin O
NADPHO: Nicotinamide adenine dinucleotide phosphate (NADPH) oxidases
ROS: Reactive oxygen species
RNI: Reactive nitrogen intermediates
