# Supplementary material for: Transcriptomic biomarker pathways associated with death in HIV-infected patients with cryptococcal meningitis
Source: BMC Med Genomics. 2021 Apr 16;14:108. doi: 10.1186/s12920-021-00914-1 (PMC8052789; doi:10.1186/s12920-021-00914-1)
Supplement: Supplementary file 5 — Additional file 5. Table S5. PLS algorithm script. Partial Least Squares (PLS) algorithm script exported from JMP14.2 Pro. [file 12920_2021_914_MOESM5_ESM.docx]

**Supplemental Table S5. PLS Script from JMP.**

Open(

"/Users/Experiments 2019/COAT. JMP14/COAT brinteracTrID-BP211_FPKM_final Common2440.jmp",

window bounds( 0, -16, 1273, 305 )

);

New Window( "COAT brinteracTrID-BP211_FPKM_final Common2440 - Fit Partial Least Squares",

Fit Model(

Y( :ART DE ),

Effects(

:Name( "CD4+" ),

:LFA,

:HIV_RNA,

:Age,

:A2M,

:ABCA1,

:ABCB10,

:ABCG4,

:ABI1,

:ABLIM1,

:ACE,

:ACO2,

:ACSL1,

:ACTB,

:ACTC1,

:ACTG1,

:ACTN1,

:ACTN4,

:ACTR2,

:ACTR3,

:ACVR1,

:ACVR1B,

:ACVR1C,

:ACVR2A,

:ACVR2B,

:ACVRL1,

:ADAM10,

:ADAM15,

:ADAM7,

:ADAM8,

:ADAMTS19,

:ADAMTS6,

:ADAP1,

:ADAP2,

:ADAR,

:ADCY1,

:ADCY2,

:ADCY6,

:ADCY7,

:ADIPOQ,

:ADORA1,

:ADORA2A,

:ADORA2B,

:ADORA3,

:ADRBK1,

:AEBP2,

:AES,

:AGAP2,

:AGL,

:AGPAT2,

:AGTR1,

:AIM1,

:AIM2,

:AIP,

:AKAP5,

:AKR1B1,

:AKR1C3,

:AKT1,

:AKT1S1,

:AKT2,

:AKT3,

:ALAS2,

:ALB,

:ALOX12,

:ALOX12B,

:ALOX12P2,

:ALOX15,

:ALOX15B,

:ALOX5,

:ALOX5AP,

:ALOXE3,

:ALPL,

:AP2A1,

:APAF1,

:APBB1IP,

:APEX1,

:APH1A,

:APH1B,

:APLNR,

:APOA2,

:APOB,

:APOBEC1,

:APOBEC2,

:APOBEC3A,

:APOBEC3B,

:APOBEC3C,

:APOBEC3D,

:APOBEC3F,

:APOBEC3G,

:APOBEC3H,

:APOBEC4,

:APOH,

:APP,

:APRT,

:AQP10,

:AQP2,

:AQP4,

:AQP6,

:AQP9,

:ARF6,

:ARG1,

:ARGLU1,

:ARHGAP1,

:ARHGAP6,

:ARHGAP9,

:ARHGDIA,

:ARHGDIB,

:ARHGEF11,

:ARHGEF12,

:ARHGEF17,

:ARHGEF2,

:ARHGEF6,

:ARHGEF9,

:ARNT,

:ARPC1A,

:ARPC1B,

:ARPC2,

:ARPC3,

:ARPC4,

:ARPC5,

:ARPC5L,

:ARRB2,

:ASB1,

:ATF2,

:ATF3,

:ATG12,

:ATG16L1,

:ATG16L2,

:ATG3,

:ATG4D,

:ATG7,

:ATG9A,

:ATM,

:ATOX1,

:ATP2A1,

:ATP5E,

:ATP5H,

:ATP5I,

:ATP5J,

:ATP5J2,

:ATP5L,

:ATP5O,

:ATP6AP1,

:ATP6AP1L,

:ATP6V0A1,

:ATP6V0A2,

:ATP6V0A4,

:ATP6V0B,

:ATP6V0C,

:ATP6V0D1,

:ATP6V0D2,

:ATP6V0E1,

:ATP6V0E2,

:ATP6V1A,

:ATP6V1B1,

:ATP6V1B2,

:ATP6V1C1,

:ATP6V1C2,

:ATP6V1D,

:ATP6V1E1,

:ATP6V1E2,

:ATP6V1F,

:ATP6V1G1,

:ATP6V1H,

:ATP9B,

:ATPIF1,

:AVP,

:AVPR2,

:B2M,

:B3GAT1,

:B4GALT1,

:BACE1,

:BACE2,

:BAD,

:BAG4,

:BAK1,

:BATF,

:BATF2,

:BATF3,

:BAX,

:BCL10,

:BCL11A,

:BCL11B,

:BCL2,

:BCL2A1,

:BCL2L1,

:BCL2L10,

:BCL2L11,

:BCL2L12,

:BCL2L13,

:BCL2L14,

:BCL2L15,

:BCL2L2,

:BCL3,

:BCL6,

:BCL6B,

:BCL7A,

:BCL7B,

:BCL7C,

:BCL9,

:BCL9L,

:BCLAF1,

:BCR,

:BDKRB2,

:BET1L,

:BEX1,

:BID,

:BIRC2,

:BLNK,

:BMI1,

:BMP6,

:BMX,

:BPI,

:BST1,

:BST2,

:BTG2,

:BTK,

:BTLA,

:BTRC,

:C1QA,

:C1QB,

:C1QBP,

:C1QC,

:C1QL1,

:C1QL2,

:C1QL3,

:C1QL4,

:C1QTNF1,

:C1QTNF2,

:C1QTNF3,

:C1QTNF4,

:C1QTNF5,

:C1QTNF6,

:C1QTNF7,

:C1QTNF8,

:C1QTNF9,

:C1QTNF9B,

:C1R,

:C1RL,

:C1S,

:C2,

:C3,

:C3AR1,

:C4A,

:C4B,

:C4BPA,

:C4BPB,

:C5,

:C5AR1,

:CABIN1,

:CACNA1E,

:CACNB3,

:CACNG4,

:CACNG7,

:CALM1,

:CALM2,

:CALM3,

:CALR,

:CALR3,

:CAMK1,

:CAMK2G,

:CAMKK2,

:CANX,

:CAP1,

:CAPG,

:CAPN1,

:CAPN2,

:CAPZA2,

:CARD10,

:CARD11,

:CARD14,

:CARD16,

:CARD17,

:CARD18,

:CARD6,

:CARD8,

:CARD9,

:CARM1,

:CASP1,

:CASP10,

:CASP12,

:CASP14,

:CASP2,

:CASP3,

:CASP4,

:CASP5,

:CASP6,

:CASP7,

:CASP8,

:CASP8AP2,

:CASP9,

:CAST,

:CBL,

:CBLB,

:CCDC14,

:CCDC42,

:CCL1,

:CCL11,

:CCL13,

:CCL14,

:CCL15,

:CCL16,

:CCL17,

:CCL18,

:CCL19,

:CCL2,

:CCL20,

:CCL21,

:CCL22,

:CCL23,

:CCL24,

:CCL25,

:CCL26,

:CCL27,

:CCL28,

:CCL3,

:CCL3L1,

:CCL3L3,

:CCL4,

:CCL4L1,

:CCL4L2,

:CCL5,

:CCL7,

:CCL8,

:CCNA1,

:CCND3,

:CCNE2,

:CCNK,

:CCR1,

:CCR10,

:CCR2,

:CCR3,

:CCR4,

:CCR5,

:CCR6,

:CCR7,

:CCR8,

:CCR9,

:CD109,

:CD14,

:CD151,

:CD160,

:CD163,

:CD163L1,

:CD164,

:CD164L2,

:CD177,

:CD180,

:CD19,

:CD1A,

:CD1B,

:CD1C,

:CD1D,

:CD1E,

:CD2,

:CD200,

:CD200R1,

:CD200R1L,

:CD207,

:CD209,

:CD22,

:CD226,

:CD24,

:CD244,

:CD247,

:CD248,

:CD27,

:CD274,

:CD276,

:CD28,

:CD2AP,

:CD2BP2,

:CD300A,

:CD300C,

:CD300E,

:CD300LB,

:CD300LD,

:CD300LF,

:CD300LG,

:CD302,

:CD320,

:CD33,

:CD34,

:CD36,

:CD37,

:CD38,

:CD3D,

:CD3E,

:CD3EAP,

:CD3G,

:CD4,

:CD40,

:CD40LG,

:CD44,

:CD46,

:CD47,

:CD48,

:CD5,

:CD52,

:CD53,

:CD55,

:CD58,

:CD59,

:CD5L,

:CD6,

:CD63,

:CD68,

:CD69,

:CD7,

:CD70,

:CD72,

:CD74,

:CD79A,

:CD79B,

:CD80,

:CD81,

:CD82,

:CD83,

:CD84,

:CD86,

:CD8A,

:CD8B,

:CD9,

:CD93,

:CD96,

:CD99,

:CD99L2,

:CDA,

:CDC40,

:CDC42,

:CDH1,

:CDH11,

:CDH12,

:CDH13,

:CDH19,

:CDH20,

:CDH24,

:CDH26,

:CDH3,

:CDH4,

:CDH6,

:CDH8,

:CDH9,

:CDK6,

:CDK9,

:CDKN1B,

:CDKN2A,

:CDS1,

:CDS2,

:CEACAM1,

:CEACAM16,

:CEACAM18,

:CEACAM19,

:CEACAM20,

:CEACAM21,

:CEACAM3,

:CEACAM4,

:CEACAM5,

:CEACAM6,

:CEACAM7,

:CEACAM8,

:CEBPA,

:CEBPB,

:CEP57,

:CERK,

:CETP,

:CFC1B,

:CFD,

:CFH,

:CFL1,

:CFLAR,

:CFP,

:CHD3,

:CHI3L1,

:CHI3L2,

:CHIA,

:CHIC1,

:CHIC2,

:CHID1,

:CHIT1,

:CHMP1A,

:CHMP1B,

:CHMP2A,

:CHMP2B,

:CHMP4A,

:CHMP4B,

:CHMP4C,

:CHPT1,

:CHRM1,

:CHRM2,

:CHRM3,

:CHRNA1,

:CHRNA3,

:CHRNA4,

:CHRNA5,

:CHRNA9,

:CHRNB3,

:CHST12,

:CHST13,

:CHST7,

:CHUK,

:CIAO1,

:CIITA,

:CISD2,

:CISH,

:CKAP4,

:CLDN2,

:CLDN5,

:CLDND1,

:CLDND2,

:CLEC10A,

:CLEC11A,

:CLEC12A,

:CLEC12B,

:CLEC14A,

:CLEC16A,

:CLEC17A,

:CLEC18C,

:CLEC1A,

:CLEC1B,

:CLEC2A,

:CLEC2B,

:CLEC2D,

:CLEC2L,

:CLEC3A,

:CLEC3B,

:CLEC4A,

:CLEC4C,

:CLEC4D,

:CLEC4E,

:CLEC4F,

:CLEC4G,

:CLEC4GP1,

:CLEC4M,

:CLEC5A,

:CLEC6A,

:CLEC7A,

:CLEC9A,

:CLECL1,

:CLGN,

:CLIP4,

:CLK1,

:CLN3,

:CLNS1A,

:CLOCK,

:CLTA,

:CLTC,

:CLU,

:CMTM2,

:CNGB1,

:CNGB3,

:CNN2,

:COG2,

:COL17A1,

:COL1A1,

:COLEC10,

:COLEC11,

:COLEC12,

:CORO1A,

:COX15,

:COX17,

:COX5B,

:COX6A1,

:COX6B1,

:COX6C,

:COX7A2,

:COX7B,

:COX7C,

:CPOX,

:CPSF6,

:CR1,

:CR1L,

:CR2,

:CRADD,

:CREB1,

:CREB3,

:CREB3L4,

:CREB5,

:CREBBP,

:CRIM1,

:CRP,

:CRYZ,

:CSF1,

:CSF1R,

:CSF2,

:CSF2RA,

:CSF2RB,

:CSF3,

:CSF3R,

:CSK,

:CSPP1,

:CSTB,

:CTLA4,

:CTSA,

:CTSB,

:CTSC,

:CTSD,

:CTSE,

:CTSF,

:CTSG,

:CTSH,

:CTSK,

:CTSO,

:CTSS,

:CTSW,

:CTSZ,

:CUL1,

:CUL4A,

:CUL5,

:CX3CR1,

:CXCL1,

:CXCL10,

:CXCL11,

:CXCL12,

:CXCL13,

:CXCL14,

:CXCL16,

:CXCL17,

:CXCL2,

:CXCL3,

:CXCL5,

:CXCL6,

:CXCL8,

:CXCL9,

:CXCR1,

:CXCR3,

:CXCR4,

:CXCR5,

:CXCR6,

:CYB5A,

:CYBB,

:CYBRD1,

:CYCS,

:CYP19A1,

:CYP1B1,

:CYP7A1,

:CYSLTR1,

:CYSLTR2,

:DAP,

:DAPK2,

:DBN1,

:DCTN2,

:DCTN4,

:DCXR,

:DDB1,

:DDIT3,

:DDX10,

:DDX3X,

:DDX54,

:DDX58,

:DDX59,

:DEFA1,

:DEFA1B,

:DEFA3,

:DEFA4,

:DEFA5,

:DEFA6,

:DEFB1,

:DEFB103A,

:DEFB103B,

:DEFB104A,

:DEFB104B,

:DEFB105A,

:DEFB105B,

:DEFB106A,

:DEFB106B,

:DEFB107A,

:DEFB107B,

:DEFB108B,

:DEFB109P1,

:DEFB110,

:DEFB112,

:DEFB113,

:DEFB114,

:DEFB115,

:DEFB116,

:DEFB118,

:DEFB119,

:DEFB121,

:DEFB122,

:DEFB123,

:DEFB124,

:DEFB125,

:DEFB126,

:DEFB127,

:DEFB128,

:DEFB129,

:DEFB130,

:DEFB131,

:DEFB132,

:DEFB133,

:DEFB134,

:DEFB135,

:DFFA,

:DFFB,

:DGKQ,

:DHX57,

:DHX58,

:DHX8,

:DHX9,

:DIABLO,

:DIAPH1,

:DIRAS3,

:DIS3,

:DIS3L,

:DIS3L2,

:DLL1,

:DLL3,

:DLL4,

:DLST,

:DNM2,

:DNMT1,

:DPEP3,

:DSP,

:DTX4,

:DUSP1,

:DUSP12,

:DYNC1H1,

:DYNC1I1,

:DYNC1I2,

:DYNC1LI1,

:DYNC1LI2,

:DYNC2H1,

:DYNLL1,

:DYNLRB1,

:DYNLRB2,

:DYNLT1,

:DYRK3,

:E4F1,

:EAF1,

:ECHDC2,

:EEA1,

:EEF1A1,

:EEF1B2,

:EGF,

:EGFR,

:EGLN1,

:EGR1,

:EIF2A,

:EIF2AK2,

:EIF2B4,

:EIF2B5,

:EIF2S1,

:EIF2S2,

:EIF2S3,

:EIF3A,

:EIF3D,

:EIF3E,

:EIF3F,

:EIF3G,

:EIF3H,

:EIF3I,

:EIF3L,

:EIF3M,

:EIF4A1,

:EIF4A2,

:EIF4B,

:EIF4E,

:EIF4EBP2,

:EIF4G1,

:EIF4G2,

:EIF4G3,

:EIF5B,

:ELF4,

:ELK1,

:EME1,

:ENPP2,

:ENTPD1,

:EOMES,

:EP300,

:EP400,

:EPHA1,

:EPHA4,

:EPHA6,

:EPHA7,

:EPHA8,

:EPHB1,

:EPHB2,

:EPHB4,

:EPS15,

:ESF1,

:ESR1,

:ETV6,

:EVL,

:EXOC7,

:EXOC8,

:EXOSC1,

:EXOSC10,

:EXOSC2,

:EXOSC3,

:EXOSC4,

:EXOSC5,

:EXOSC6,

:EXOSC7,

:EXOSC8,

:EXOSC9,

:F11R,

:F2,

:F2R,

:F2RL1,

:F2RL2,

:F2RL3,

:F8,

:FABP2,

:FADD,

:FAM153B,

:FAM172A,

:FAM179A,

:FAM188A,

:FAM65B,

:FAM72A,

:FAM72B,

:FAM76B,

:FAM96A,

:FAS,

:FASLG,

:FASN,

:FAU,

:FBXL5,

:FBXO30,

:FBXO36,

:FBXO6,

:FBXO7,

:FBXW11,

:FCER1A,

:FCER1G,

:FCGBP,

:FCGR1A,

:FCGR1B,

:FCGR2A,

:FCGR2B,

:FCGR2C,

:FCGR3A,

:FCGR3B,

:FCGRT,

:FCN1,

:FCN2,

:FCN3,

:FCRL1,

:FCRL2,

:FCRL3,

:FCRL4,

:FECH,

:FERMT2,

:FERMT3,

:FGA,

:FGB,

:FGFBP2,

:FGFR1,

:FGFR1OP,

:FGFR1OP2,

:FGFR2,

:FGFR3,

:FGFR4,

:FGG,

:FGR,

:FHL1,

:FHL2,

:FHL3,

:FHL5,

:FLII,

:FLNA,

:FLT1,

:FLT3LG,

:FN1,

:FNBP1,

:FNTA,

:FOLR3,

:FOS,

:FOXO1,

:FOXO3,

:FOXO4,

:FOXO6,

:FOXP1,

:FOXP2,

:FOXP3,

:FOXP4,

:FPR1,

:FPR2,

:FRS2,

:FTH1,

:FTL,

:FUT7,

:FYB,

:FYN,

:FZD1,

:GAA,

:GAB1,

:GAB2,

:GABPA,

:GABPB2,

:GABRR2,

:GAD2,

:GADD45B,

:GANAB,

:GATA1,

:GATA2,

:GATA3,

:GATA4,

:GATA5,

:GATA6,

:GBP1,

:GCA,

:GDI1,

:GFI1,

:GGA2,

:GIMAP2,

:GIT2,

:GK,

:GLG1,

:GLI1,

:GLIPR2,

:GLRX5,

:GLS,

:GLUL,

:GMEB1,

:GMEB2,

:GMFB,

:GMFG,

:GMPS,

:GNA11,

:GNA12,

:GNA13,

:GNAI2,

:GNAI3,

:GNAL,

:GNAQ,

:GNAS,

:GNAZ,

:GNB1,

:GNB1L,

:GNB2,

:GNB4,

:GNG10,

:GNG11,

:GNG12,

:GNG2,

:GNG3,

:GNG4,

:GNG5,

:GNGT2,

:GNL3,

:GNL3L,

:GNLY,

:GNRH1,

:GOPC,

:GORASP1,

:GOSR1,

:GOSR2,

:GOT2,

:GP2,

:GP5,

:GP6,

:GP9,

:GPAA1,

:GPR18,

:GPR83,

:GRAP2,

:GRB2,

:GRIA1,

:GRIN2C,

:GRIP1,

:GRK6,

:GSAP,

:GSDMD,

:GSK3A,

:GSK3B,

:GSN,

:GSR,

:GSTM1,

:GSTM2,

:GSTM4,

:GUCY1A2,

:GYPB,

:GZMA,

:GZMB,

:GZMH,

:GZMK,

:GZMM,

:H3F3A,

:HAVCR1,

:HAVCR2,

:HCK,

:HCLS1,

:HCST,

:HDAC1,

:HDAC10,

:HDAC2,

:HDAC3,

:HDAC7,

:HDAC8,

:HERC1,

:HGF,

:HGS,

:HIF1A,

:HIST1H2BO,

:Name( "HLA-A" ),

:Name( "HLA-B" ),

:Name( "HLA-C" ),

:Name( "HLA-DMA" ),

:Name( "HLA-DMB" ),

:Name( "HLA-DOA" ),

:Name( "HLA-DOB" ),

:Name( "HLA-DPA1" ),

:Name( "HLA-DPB1" ),

:Name( "HLA-DPB2" ),

:Name( "HLA-DQA1" ),

:Name( "HLA-DQA2" ),

:Name( "HLA-DQB1" ),

:Name( "HLA-DQB2" ),

:Name( "HLA-DRA" ),

:Name( "HLA-DRB1" ),

:Name( "HLA-DRB3" ),

:Name( "HLA-DRB4" ),

:Name( "HLA-DRB5" ),

:Name( "HLA-DRB6" ),

:Name( "HLA-E" ),

:Name( "HLA-F" ),

:Name( "HLA-G" ),

:Name( "HLA-H" ),

:HMBS,

:HMGB1,

:HMOX1,

:HNF1A,

:HNRNPA1,

:HNRNPA2B1,

:HNRNPF,

:HNRNPK,

:HRASLS5,

:HRG,

:HRH2,

:HS3ST3B1,

:HSP90B1,

:HSPA2,

:HSPA5,

:HSPA6,

:ICAM1,

:ICAM2,

:ICAM3,

:ICAM4,

:ICAM5,

:ICK,

:ICOS,

:ICOSLG,

:ID3,

:ID4,

:IDE,

:IDO1,

:IDO2,

:IFI16,

:IFI27,

:IFI27L1,

:IFI27L2,

:IFI30,

:IFI35,

:IFI44,

:IFI44L,

:IFI6,

:IFIH1,

:IFIT1,

:IFIT2,

:IFIT3,

:IFIT5,

:IFITM1,

:IFITM2,

:IFITM3,

:IFITM4P,

:IFITM5,

:IFNA1,

:IFNA10,

:IFNA13,

:IFNA14,

:IFNA16,

:IFNA17,

:IFNA2,

:IFNA21,

:IFNA4,

:IFNA5,

:IFNA6,

:IFNA7,

:IFNA8,

:IFNAR1,

:IFNAR2,

:IFNB1,

:IFNE,

:IFNG,

:IFNGR1,

:IFNGR2,

:IFNK,

:IFNLR1,

:IFRD1,

:IGF1R,

:IGF2BP2,

:IKBKE,

:IKBKG,

:IKZF2,

:IKZF3,

:IKZF4,

:IKZF5,

:IL10,

:IL10RA,

:IL10RB,

:IL11,

:IL11RA,

:IL12A,

:IL12B,

:IL12RB1,

:IL12RB2,

:IL13,

:IL13RA1,

:IL13RA2,

:IL15,

:IL15RA,

:IL16,

:IL17A,

:IL17B,

:IL17C,

:IL17D,

:IL17F,

:IL17RA,

:IL17RB,

:IL17RC,

:IL17RD,

:IL17RE,

:IL17REL,

:IL18,

:IL18BP,

:IL18R1,

:IL18RAP,

:IL19,

:IL1A,

:IL1B,

:IL1F10,

:IL1R1,

:IL1R2,

:IL1RAP,

:IL1RAPL1,

:IL1RAPL2,

:IL1RL1,

:IL1RL2,

:IL1RN,

:IL2,

:IL20,

:IL20RA,

:IL20RB,

:IL21,

:IL21R,

:IL22,

:IL22RA1,

:IL22RA2,

:IL23A,

:IL23R,

:IL24,

:IL25,

:IL26,

:IL27,

:IL27RA,

:IL2RA,

:IL2RB,

:IL2RG,

:IL3,

:IL31,

:IL31RA,

:IL32,

:IL33,

:IL34,

:IL3RA,

:IL4,

:IL4I1,

:IL4R,

:IL5,

:IL5RA,

:IL6,

:IL6R,

:IL6ST,

:IL7,

:IL7R,

:IL9,

:IL9R,

:ILF3,

:ILK,

:IMPDH2,

:INHBA,

:INSIG1,

:INTS6,

:IQCF1,

:IQCF2,

:IQCF3,

:IQCG,

:IQCH,

:IQCJ,

:IQCK,

:IQGAP1,

:IRAK1,

:IRAK1BP1,

:IRAK2,

:IRAK3,

:IRAK4,

:IREB2,

:IRF1,

:IRF2,

:IRF2BP1,

:IRF2BP2,

:IRF3,

:IRF4,

:IRF5,

:IRF6,

:IRF7,

:IRF8,

:IRF9,

:IRS2,

:ISCU,

:ISG15,

:ISG20,

:ISG20L2,

:ITFG3,

:ITGA1,

:ITGA10,

:ITGA11,

:ITGA2,

:ITGA2B,

:ITGA3,

:ITGA4,

:ITGA5,

:ITGA6,

:ITGA7,

:ITGA8,

:ITGA9,

:ITGAD,

:ITGAE,

:ITGAL,

:ITGAM,

:ITGAV,

:ITGAX,

:ITGB1,

:ITGB1BP1,

:ITGB1BP2,

:ITGB2,

:ITGB3,

:ITGB3BP,

:ITGB4,

:ITGB5,

:ITGB6,

:ITGB7,

:ITGB8,

:ITGBL1,

:ITK,

:ITM2A,

:ITM2B,

:ITM2C,

:ITPKB,

:ITPR1,

:ITPR2,

:ITPR3,

:JAG1,

:JAG2,

:JAK1,

:JAK2,

:JAK3,

:JMJD6,

:JSRP1,

:JUN,

:JUNB,

:JUND,

:KAT2A,

:KCNE3,

:KCNJ9,

:KDM6B,

:KDR,

:KIR2DL1,

:KIR2DL2,

:KIR2DL3,

:KIR2DL4,

:KIR2DL5A,

:KIR2DL5B,

:KIR2DS1,

:KIR2DS2,

:KIR2DS3,

:KIR2DS4,

:KIR2DS5,

:KIR3DL1,

:KIR3DL2,

:KIR3DL3,

:KIR3DS1,

:KIR3DX1,

:KIT,

:KLF1,

:KLF2,

:KLF6,

:KLHDC1,

:KLHDC10,

:KLHDC2,

:KLHDC3,

:KLHDC4,

:KLHDC7A,

:KLHDC7B,

:KLHDC8A,

:KLHDC8B,

:KLHDC9,

:KLHL25,

:KLK1,

:KLK8,

:KLKP1,

:KLRB1,

:KLRC1,

:KLRC2,

:KLRC3,

:KLRC4,

:KLRD1,

:KLRF1,

:KLRG1,

:KLRG2,

:KLRK1,

:KRAS,

:KRT18,

:LAG3,

:LAGE3,

:LAIR1,

:LAIR2,

:LAMB2,

:LAMP1,

:LAMP2,

:LAMP3,

:LAT,

:LATS2,

:LCK,

:LCN2,

:LCP1,

:LCP2,

:LCT,

:LGALS1,

:LGALS12,

:LGALS13,

:LGALS14,

:LGALS2,

:LGALS3,

:LGALS3BP,

:LGALS4,

:LGALS7,

:LGALS7B,

:LGALS8,

:LGALS9,

:LILRA1,

:LILRA2,

:LILRA3,

:LILRA4,

:LILRA5,

:LILRA6,

:LILRB1,

:LILRB2,

:LILRB3,

:LILRB4,

:LILRB5,

:LILRP2,

:LIME1,

:LIMK1,

:LIMK2,

:LIN7C,

:LITAF,

:LMAN1,

:LMAN1L,

:LMAN2,

:LMAN2L,

:LMO2,

:LMTK3,

:LPAR2,

:LPO,

:LRIG1,

:LRIG2,

:LRIG3,

:LRP10,

:LRPPRC,

:LRRC3,

:LSM3,

:LSM5,

:LSM6,

:LSP1,

:LTA,

:LTB,

:LTB4R,

:LTB4R2,

:LTBR,

:LTF,

:LTK,

:LY75,

:LY86,

:LY9,

:LY96,

:LYN,

:LYZ,

:M6PR,

:MAF,

:MAGI1,

:MAGI2,

:MAGI3,

:MALT1,

:MAP2K2,

:MAP2K3,

:MAP2K4,

:MAP2K6,

:MAP2K7,

:MAP3K1,

:MAP3K11,

:MAP3K14,

:MAP3K2,

:MAP3K3,

:MAP3K4,

:MAP3K5,

:MAP4K1,

:MAPK1,

:MAPK10,

:MAPK11,

:MAPK12,

:MAPK13,

:MAPK14,

:MAPK15,

:MAPK1IP1L,

:MAPK3,

:MAPK4,

:MAPK6,

:MAPK7,

:MAPK8,

:MAPK8IP1,

:MAPK8IP2,

:MAPK8IP3,

:MAPK9,

:MAPKAP1,

:MAPKAPK2,

:MAPKAPK3,

:MAPKAPK5,

:MAPKBP1,

:MARCO,

:MARK1,

:MARK2,

:MARK3,

:MARK4,

:MASP1,

:MASP2,

:MAST1,

:MATK,

:MATR3,

:MAX,

:MBL2,

:MBLAC1,

:MBLAC2,

:MBOAT1,

:MCOLN1,

:MCOLN2,

:MED14,

:MED15,

:MED26,

:MED28,

:MED31,

:MED7,

:MERTK,

:METAP2,

:MFGE8,

:MGAM,

:MGAT1,

:MIAT,

:MID1IP1,

:MIF,

:MIP,

:MIR380,

:MKI67,

:MKL1,

:MME,

:MMP1,

:MMP10,

:MMP11,

:MMP12,

:MMP13,

:MMP14,

:MMP15,

:MMP16,

:MMP17,

:MMP19,

:MMP2,

:MMP20,

:MMP21,

:MMP23A,

:MMP23B,

:MMP24,

:MMP25,

:MMP26,

:MMP27,

:MMP28,

:MMP3,

:MMP7,

:MMP8,

:MMP9,

:MMS19,

:MOSPD2,

:MPHOSPH6,

:MPO,

:MPP6,

:MPZ,

:MR1,

:MRC1,

:MRC2,

:MRPL13,

:MSN,

:MT1X,

:MT2A,

:MTAP,

:MTCH1,

:MTCH2,

:MTNR1A,

:MUS81,

:MX1,

:MX2,

:MXD1,

:MXD3,

:MXD4,

:MYADM,

:MYB,

:MYBL1,

:MYBL2,

:MYC,

:MYD88,

:MYH14,

:MYH9,

:MYL12A,

:MYL3,

:MYL4,

:MYL5,

:MYL9,

:MYO1F,

:MYO1G,

:MYO9B,

:NAIP,

:NAMPT,

:NANOS1,

:NANOS2,

:NANOS3,

:NAPA,

:NAPB,

:NAPG,

:NBEAL2,

:NCALD,

:NCAM1,

:NCF1,

:NCF2,

:NCK1,

:NCK2,

:NCOR2,

:NCR1,

:NCR2,

:NCR3,

:NCSTN,

:NDRG1,

:NDUFA1,

:NDUFA12,

:NDUFA13,

:NDUFA4,

:NDUFA5,

:NDUFA7,

:NDUFAF2,

:NDUFB1,

:NDUFB10,

:NDUFB2,

:NDUFB3,

:NDUFB4,

:NDUFB9,

:NDUFS4,

:NDUFS5,

:NDUFS6,

:NDUFS8,

:NEFL,

:NEK1,

:NEK2,

:NEK3,

:NEK4,

:NEK5,

:NEK6,

:NEK7,

:NEK8,

:NEK9,

:NELL1,

:NELL2,

:NET1,

:NFAM1,

:NFAT5,

:NFATC1,

:NFATC2,

:NFATC2IP,

:NFATC3,

:NFATC4,

:NFE2L2,

:NFIA,

:NFIL3,

:NFKB1,

:NFKB2,

:NFKBIA,

:NFKBIB,

:NFKBID,

:NFKBIE,

:NFKBIL1,

:NFKBIZ,

:NFRKB,

:NGF,

:NICN1,

:NINJ1,

:NKG7,

:NKIRAS1,

:NKIRAS2,

:NKRF,

:NKTR,

:NLRC3,

:NLRC4,

:NLRC5,

:NLRP1,

:NLRP10,

:NLRP11,

:NLRP12,

:NLRP13,

:NLRP14,

:NLRP2,

:NLRP3,

:NLRP4,

:NLRP5,

:NLRP6,

:NLRP7,

:NLRP8,

:NLRP9,

:NLRX1,

:NMUR1,

:NOD1,

:NOD2,

:NOS1,

:NOTCH1,

:NOTCH2,

:NOX1,

:NOX3,

:NOX4,

:NOX5,

:NPHP3,

:NPM1,

:NR0B1,

:NR1H3,

:NR4A1,

:NRGN,

:NRL,

:NRP1,

:NSF,

:NTRK3,

:NUBP1,

:NUDC,

:NUMB,

:NUP107,

:NUP62,

:OAS1,

:OAS2,

:OAS3,

:OASL,

:OLFM4,

:OLR1,

:ORAI1,

:ORAI2,

:ORM1,

:ORM2,

:OSM,

:OSMR,

:OTOF,

:OTUD1,

:OXR1,

:P2RX1,

:P2RX7,

:PAAF1,

:PABPC4,

:PAFAH1B1,

:PAG1,

:PAK1,

:PAK2,

:PAK3,

:PANK3,

:PANX1,

:PANX2,

:PANX3,

:PARP1,

:PARP14,

:PARP15,

:PARP9,

:PARVB,

:PCBP1,

:PCYOX1,

:PDCD1,

:PDCD10,

:PDCD11,

:PDCD1LG2,

:PDCD2,

:PDCD2L,

:PDCD4,

:PDCD5,

:PDCD6,

:PDCD6IP,

:PDCD7,

:PDCL,

:PDCL2,

:PDCL3,

:PDDC1,

:PDGFA,

:PDGFC,

:PDGFD,

:PDGFRA,

:PDGFRB,

:PDK1,

:PDPK1,

:PELI1,

:PELI2,

:PELI3,

:PFDN5,

:PFKL,

:PFN1,

:PGLYRP1,

:PGLYRP2,

:PGM1,

:PGM2,

:PGM2L1,

:PGM3,

:PGM5,

:PHF12,

:PHF2,

:PHF5A,

:PI3,

:PI4KA,

:PIAS1,

:PIAS2,

:PIAS3,

:PIAS4,

:PIGK,

:PIK3AP1,

:PIK3C2A,

:PIK3C2B,

:PIK3C2G,

:PIK3C3,

:PIK3CA,

:PIK3CB,

:PIK3CD,

:PIK3CG,

:PIK3IP1,

:PIK3R1,

:PIK3R2,

:PIK3R3,

:PIK3R4,

:PIK3R5,

:PIK3R6,

:PIKFYVE,

:PIP4K2C,

:PIP5K1A,

:PIP5K1B,

:PIP5K1C,

:PLA2G1B,

:PLA2G2D,

:PLA2G4B,

:PLA2G4D,

:PLA2G4E,

:PLA2G4F,

:PLA2G5,

:PLA2G7,

:PLA2R1,

:PLCB2,

:PLCG1,

:PLCG2,

:PLD1,

:PLEKHM1,

:PLXNC1,

:PML,

:PMP22,

:PNPLA6,

:POLG,

:POLR2C,

:POLR2I,

:POLR2J,

:POLR2K,

:PPARG,

:PPBP,

:PPIA,

:PPM1A,

:PPM1J,

:PPOX,

:PPP1CC,

:PPP1R12A,

:PPP1R12C,

:PPP1R15A,

:PPP2CA,

:PPP2R1A,

:PPP2R2A,

:PPP2R2B,

:PPP2R4,

:PPP2R5B,

:PPP2R5C,

:PPP2R5E,

:PPP3CC,

:PPP3R1,

:PPY,

:PQBP1,

:PRCP,

:PRDM2,

:PRDM4,

:PRDM5,

:PRDM6,

:PRDM7,

:PRDM8,

:PRDM9,

:PRDX1,

:PRDX2,

:PRDX5,

:PRDX6,

:PREX1,

:PREX2,

:PRF1,

:PRKAA1,

:PRKAA2,

:PRKACA,

:PRKACB,

:PRKACG,

:PRKAG2,

:PRKAR1A,

:PRKAR1B,

:PRKAR2B,

:PRKCA,

:PRKCB,

:PRKCD,

:PRKCDBP,

:PRKCE,

:PRKCG,

:PRKCH,

:PRKCI,

:PRKCQ,

:PRKCSH,

:PRKCZ,

:PRKD1,

:PRKG1,

:PRKX,

:PRNP,

:PROK2,

:PRPF18,

:PRPF3,

:PRPF38A,

:PRPF38B,

:PRPF39,

:PRPF4,

:PRPF4B,

:PRTN3,

:PSEN1,

:PSEN2,

:PSENEN,

:PSIP1,

:PSMA2,

:PSMB8,

:PSMB9,

:PSMC3,

:PSME2,

:PTAFR,

:PTEN,

:PTGDR,

:PTGDS,

:PTGER1,

:PTGER2,

:PTGER3,

:PTGER4,

:PTGES,

:PTGES2,

:PTGES3,

:PTGFR,

:PTGIS,

:PTGS2,

:PTK2B,

:PTK7,

:PTPN1,

:PTPN11,

:PTPN2,

:PTPN22,

:PTPN6,

:PTPN7,

:PTPRC,

:PTPRH,

:PTPRJ,

:PTRF,

:PTX3,

:PVRL2,

:PXN,

:PYCARD,

:PYDC1,

:PYGB,

:PYGL,

:PYGM,

:RAB11A,

:RAB27A,

:RAB2A,

:RAB3D,

:RAB5A,

:RAB5B,

:RAB5C,

:RAB7A,

:RAB7B,

:RAB8A,

:RAB8B,

:RAC1,

:RAC2,

:RAF1,

:RALB,

:RANBP2,

:RAP1B,

:RAP1GAP,

:RAP2A,

:RAPGEF1,

:RAPGEF3,

:RARA,

:RARRES3,

:RASAL3,

:RASGRP1,

:RASGRP4,

:RBM10,

:RBM19,

:RBM25,

:RBM7,

:RBP1,

:RDX,

:REL,

:RELA,

:RELB,

:REPIN1,

:RETNLB,

:RGL1,

:RGS18,

:RGS3,

:RGS7,

:RHOA,

:RHOB,

:RHOBTB2,

:RHOC,

:RHOF,

:RHOG,

:RHOT1,

:RHOT2,

:RHOU,

:RHOV,

:RILP,

:RIOK3,

:RIPK1,

:RIPK2,

:RNASEL,

:RND2,

:RND3,

:RNF10,

:RNF103,

:RNF11,

:RNF111,

:RNF112,

:RNF113A,

:RNF113B,

:RNF114,

:RNF115,

:RNF121,

:RNF122,

:RNF123,

:RNF125,

:RNF126,

:RNF126P1,

:RNF128,

:RNF13,

:RNF130,

:RNF133,

:RNF135,

:RNF138,

:RNF138P1,

:RNF139,

:RNF14,

:RNF141,

:RNF144A,

:RNF144B,

:RNF145,

:RNF146,

:RNF148,

:RNF149,

:RNF150,

:RNF151,

:RNF152,

:RNF157,

:RNF165,

:RNF166,

:RNF167,

:RNF168,

:RNF169,

:RNF17,

:RNF170,

:RNF175,

:RNF180,

:RNF181,

:RNF182,

:RNF183,

:RNF185,

:RNF186,

:RNF187,

:RNF19A,

:RNF19B,

:RNF2,

:RNF20,

:RNF207,

:RNF208,

:RNF212,

:RNF213,

:RNF214,

:RNF215,

:RNF216,

:RNF217,

:RNF219,

:RNF220,

:RNF24,

:RNF25,

:RNF26,

:RNF31,

:RNF32,

:RNF34,

:RNF38,

:RNF39,

:RNF4,

:RNF40,

:RNF41,

:RNF43,

:RNF44,

:RNF5,

:RNF5P1,

:RNF6,

:RNF7,

:RNF8,

:RNPC3,

:Name( "RNU4-1" ),

:Name( "RNU4-2" ),

:ROBO3,

:ROCK1,

:ROCK2,

:RPS6KA1,

:RPS6KB1,

:RPS6KB2,

:RPS7,

:RPS8,

:RPSA,

:RPTOR,

:RRAS2,

:RRP1,

:RRP12,

:RRP15,

:RRP1B,

:RRP7A,

:RRP8,

:RRP9,

:RSAD1,

:RSAD2,

:RSL1D1,

:RSL24D1,

:RSU1,

:RTN4,

:RUNX3,

:RXRA,

:S100A7,

:S100A9,

:S1PR1,

:S1PR2,

:S1PR3,

:S1PR4,

:S1PR5,

:SAMHD1,

:SAP30BP,

:SAT1,

:SCAF1,

:SCARA3,

:SCARA5,

:SCARB1,

:SCARB2,

:SCARF1,

:SCARF2,

:SCD,

:SDF4,

:SDHA,

:SEC61B,

:SEC61G,

:SELE,

:SELENBP1,

:SELL,

:SELP,

:SELPLG,

:SEMA4A,

:SENP6,

:SERPINA1,

:SERPINA3,

:SERPINB1,

:SERPINB6,

:SERPINE1,

:SERPINF1,

:SESN3,

:SETD1B,

:SF3A1,

:SF3B1,

:SFT2D1,

:SGK1,

:SH2D1A,

:SH2D3C,

:SH3BP2,

:SHB,

:SHC1,

:SHC2,

:SHMT2,

:SIDT2,

:SIGIRR,

:SIGLEC1,

:SIGLEC10,

:SIGLEC11,

:SIGLEC12,

:SIGLEC14,

:SIGLEC15,

:SIGLEC16,

:SIGLEC5,

:SIGLEC6,

:SIGLEC7,

:SIGLEC8,

:SIGLEC9,

:SIRPA,

:SIRT2,

:SKAP1,

:SKP2,

:SLAMF1,

:SLAMF6,

:SLAMF7,

:SLAMF8,

:SLAMF9,

:SLC10A1,

:SLC1A3,

:SLC25A24,

:SLC25A37,

:SLC27A4,

:SLC35B2,

:SLC35C2,

:SLC40A1,

:SLC48A1,

:SLC7A5,

:SLFN11,

:SLFN12,

:SLFN12L,

:SLITRK1,

:SLPI,

:SMAD3,

:SMPD1,

:SNAP25,

:SNCA,

:SNRNP27,

:SNRNP35,

:SNRNP70,

:SNRPA,

:SNRPA1,

:SNRPB2,

:SNRPC,

:SNRPD2,

:SNRPD3,

:SNRPF,

:SNRPG,

:SOCS1,

:SOCS2,

:SOCS3,

:SOCS4,

:SOCS5,

:SOCS6,

:SOCS7,

:SOD1,

:SOD2,

:SOS1,

:SOS2,

:SP1,

:SP3,

:SPAST,

:SPI1,

:SPTAN1,

:SRA1,

:SRC,

:SRF,

:SRXN1,

:SSR1,

:ST14,

:ST6GAL1,

:ST6GAL2,

:ST6GALNAC2,

:STAG3L1,

:STARD3NL,

:STAT1,

:STAT2,

:STAT3,

:STAT4,

:STAT5A,

:STAT5B,

:STAT6,

:STEAP3,

:STK4,

:STS,

:STUB1,

:STX11,

:STX16,

:SUB1,

:SUCLA2,

:SUCLG2,

:SULT1B1,

:SULT4A1,

:SUMO1,

:SUMO2,

:SYK,

:SYTL2,

:SYVN1,

:TAF15,

:TANK,

:TAP1,

:TAP2,

:TAPBP,

:TAPBPL,

:TARP,

:TATDN1,

:TBK1,

:TBKBP1,

:TBL1X,

:TBX21,

:TCEB1,

:TCF7,

:TCIRG1,

:TDRD7,

:TEK,

:TFR2,

:TFRC,

:TGFB1,

:TGFB1I1,

:TGFB3,

:TGFBI,

:TGFBR3,

:TGM1,

:TGM2,

:THBD,

:THUMPD1,

:TICAM1,

:TICAM2,

:TIGIT,

:TIMD4,

:TIMM10,

:TIMP2,

:TIPARP,

:TIRAP,

:TLN1,

:TLR1,

:TLR10,

:TLR2,

:TLR3,

:TLR4,

:TLR5,

:TLR6,

:TLR7,

:TLR8,

:TLR9,

:TMED2,

:TMEM120A,

:TMEM123,

:TMEM126B,

:TMEM127,

:TMEM173,

:TMEM50A,

:TMEM63A,

:TMSB4Y,

:TMX1,

:TNF,

:TNFAIP1,

:TNFAIP2,

:TNFAIP3,

:TNFAIP6,

:TNFAIP8,

:TNFAIP8L1,

:TNFAIP8L2,

:TNFAIP8L3,

:TNFRSF10A,

:TNFRSF10B,

:TNFRSF10C,

:TNFRSF10D,

:TNFRSF11A,

:TNFRSF11B,

:TNFRSF12A,

:TNFRSF13B,

:TNFRSF13C,

:TNFRSF14,

:TNFRSF17,

:TNFRSF18,

:TNFRSF19,

:TNFRSF1A,

:TNFRSF1B,

:TNFRSF21,

:TNFRSF25,

:TNFRSF4,

:TNFRSF6B,

:TNFRSF8,

:TNFRSF9,

:TNFSF10,

:TNFSF11,

:TNFSF12,

:Name( "TNFSF12-TNFSF13" ),

:TNFSF13,

:TNFSF13B,

:TNFSF14,

:TNFSF15,

:TNFSF18,

:TNFSF4,

:TNFSF8,

:TNFSF9,

:TNIP3,

:TOMM20,

:TOP2B,

:TPO,

:TPPP2,

:TRABD,

:TRADD,

:TRAF1,

:TRAF2,

:TRAF3,

:TRAF3IP1,

:TRAF3IP2,

:TRAF3IP3,

:TRAF4,

:TRAF5,

:TRAF6,

:TRAF7,

:TRAM1,

:TRAT1,

:TREM1,

:TREM2,

:TREML1,

:TREML2,

:TREML4,

:TREX1,

:TRIM14,

:TRIM21,

:TRIM25,

:TRIM44,

:TRIM5,

:TRIM56,

:TRIM58,

:TRIP12,

:TRRAP,

:TSG101,

:TSLP,

:TTR,

:TUBA1A,

:TUBA1B,

:TUBA1C,

:TUBA3E,

:TUBA4A,

:TUBA8,

:TUBB1,

:TUBB2A,

:TUBB2B,

:TUBB3,

:TUBB6,

:TUBB8,

:TUBD1,

:TUBE1,

:TUBG1,

:TUBG2,

:TWSG1,

:TXNDC12,

:TXNDC9,

:TYK2,

:TYMP,

:TYROBP,

:U2AF1,

:UBE2Z,

:UCKL1,

:UNC119,

:UNC119B,

:UNC93A,

:UNC93B1,

:UQCR10,

:UQCR11,

:UQCRB,

:UQCRH,

:UTP11L,

:UTP6,

:VAMP3,

:VASP,

:VAV1,

:VAV2,

:VAV3,

:VCAM1,

:VEGFA,

:VEGFB,

:VEGFC,

:VIM,

:VIPR2,

:VPS11,

:VPS16,

:VPS18,

:VPS28,

:VPS33A,

:VPS33B,

:VPS37A,

:VPS37B,

:VPS37C,

:VPS39,

:VPS41,

:VPS4A,

:VPS4B,

:VTA1,

:VTI1A,

:VTI1B,

:VTN,

:VWCE,

:VWDE,

:WAS,

:WASF1,

:WASL,

:WDR18,

:WIPF1,

:WNT1,

:XAF1,

:XIAP,

:XPC,

:XRCC6,

:YBX1,

:YKT6,

:YTHDC2,

:ZAP70,

:ZBP1,

:ZBTB2,

:ZCCHC17,

:ZEB1,

:ZMAT5,

:ZMPSTE24,

:ZNF148,

:ZNF182,

:ZNF254,

:ZNF292,

:ZNF398,

:ZNF668,

:ZNF791,

:ZYX

),

No Intercept( 1 ),

Center Polynomials( 0 ),

Personality( "Partial Least Squares" ),

Standardize X( 0 ),

Run(

Initial Number of Factors( 15 ),

Validation Method( Name( "Leave-One-Out" ), Initial Number of Factors( 15 ) ),

Fit( Method( NIPALS ), Number of Factors( 14 ) )

),

SendToReport(

Dispatch(

{"NIPALS Fit with 14 Factors"},

"Model Coefficients for Centered and Scaled Data",

OutlineBox,

{Close( 0 )}

),

Dispatch(

{"NIPALS Fit with 14 Factors"},

"Model Coefficients for Original Data",

OutlineBox,

{Close( 0 )}

)

)

)

) << Move Window( 0, -44 ) << Size Window( 1281, 734 ) << Maximize Display << Set Window Icon( "Model" );

<<Show Window( 0 );
