# Supplementary material for: Transcriptomic biomarker pathways associated with death in HIV-infected patients with cryptococcal meningitis
Source: BMC Med Genomics. 2021 Apr 16;14:108. doi: 10.1186/s12920-021-00914-1 (PMC8052789; doi:10.1186/s12920-021-00914-1)
Supplement: Supplementary file 6 — Additional file 6. Figure S1a, S1b. The fold change expression values for up- and down-regulated transcripts in C-IRIS Survivor Group, as compared to NoC-IRIS or Death Group. S1a. The low antiviral gene expressions at the time of antiretroviral therapy (ART) initiation in cryptococcosis-associated immune reconstitution inflammatory syndrome (C-IRIS) group. S1b. An example of upregulated transcripts in C-IRIS Survivor Group, as compared to No C-IRIS or Death Group, at week 0. [file 12920_2021_914_MOESM6_ESM.docx]

**Supplemental Figure S1.**

The fold change expression values for the Interferon-STAT and antiviral defense pathways and selected upregulated transcripts in C-IRIS Survivor Group, as compared to No C-IRIS or Death Group.

**1a.** The low antiviral gene expressions at the time of antiretroviral therapy (ART) initiation in cryptococcosis-associated immune reconstitution inflammatory syndrome (C-IRIS) group.

C-IRIS survivors group exhibited significantly lower expression of genes associated with innate immune responses: specifically, those that involve activation of antiviral defense (for example, interferon-inducible genes (IFI), and other, connected with orange lines), or genes encoding enzymes, that directly restrict or inhibit viral

replication (for example, oligoadenylate synthase [OAS] or helicases [DHX]). The transcription of interferon-induced protein with tetratricopeptide repeats (IFIT) family genes is predominantly activated by type I and type III interferons and transmitted by the pattern recognition and the JAK-STAT signaling pathways. The OAS genes encode a synthase family that is induced by interferons and catalyze the 2’,5’-oligomers of adenosine to bind and activate RNase L. The IFIT and OAS families of genes are one of many interferon-stimulated genes that play a significant role in the inhibition of cellular protein synthesis in infected cells and is particularly important in cellular resistance to viral infections.

Transcripts (nodes), colored in blue, were downregulated (>1.5-fold). Fold change for these transcripts’ expression is shown under the nodes. The orange line represents activation effect of the encoded protein. The blue line represents inactivation effect of the encoded protein. Transcripts were identified through functional analysis using Ingenuity Pathway Assistant (IPA) software.





**1b.** An example of upregulated transcripts C-IRIS Survivor Group, as compared to No C-IRIS or Death Group, at week 0.


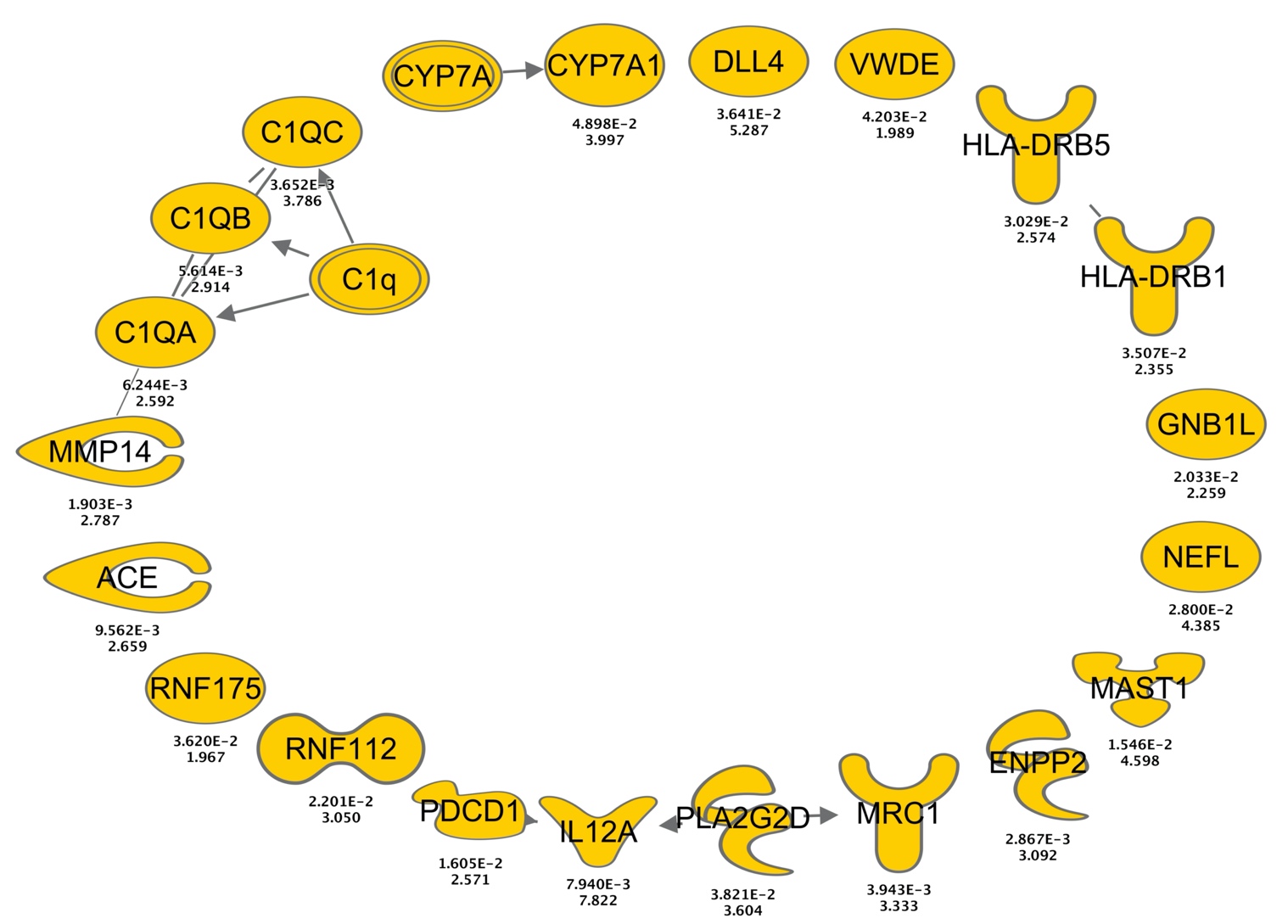


Top number, p-value;

Bottom number, fold change in expression.

Legend:

ACE, angiotensin I converting enzyme

C1q, complement C1q family

C1QA, complement C1q A chain

C1QB, complement C1q B chain

C1QC, complement C1q C chain

CYP7A,

CYP7A1, cytochrome P450 family 7 subfamily A member 1

DLL4, delta like canonical Notch ligand 4

ENPP2, ectonucleotide pyrophosphatase/phosphodiesterase 2

GNB1L, G protein subunit beta 1 like

HLA-DRB1, major histocompatibility complex, class II, DR beta 1

HLA-DRB5, major histocompatibility complex, class II, DR beta 5

IL12A, interleukin 12A

MAST1, microtubule associated serine/threonine kinase 1

MMP14, matrix metallopeptidase 14

MRC1, mannose receptor C-type 1

NEFL, neurofilament light

PDCD1, programmed cell death 1

PLA2G2D, phospholipase A2 group IID

RNF112, ring finger protein 112

RNF175, ring finger protein 175

VWDE, von Willebrand factor D and EGF domains.
